# Transcriptomic analysis reveals key factors in fruit ripening and rubbery texture caused by 1-MCP in papaya

**DOI:** 10.1186/s12870-019-1904-x

**Published:** 2019-07-12

**Authors:** Xiaoyang Zhu, Lanlan Ye, Xiaochun Ding, Qiyang Gao, Shuangling Xiao, Qinqin Tan, Jiling Huang, Weixin Chen, Xueping Li

**Affiliations:** State Key Laboratory for Conservation and Utilization of Subtropical Agro-Bioresources/Guangdong Provincial Key Laboratory of Postharvest Science of Fruits and Vegetables, College of Horticulture, South China Agricultural University, Guangzhou, 510642 Guangdong China

**Keywords:** Papaya, 1-MCP, Ethylene, Rubbery texture, Differentially expressed genes, Transcriptome, Cell wall, Cellulose and lignin

## Abstract

**Background:**

Ethylene promotes fruit ripening whereas 1-methylcyclopropene (1-MCP), a non-toxic antagonist of ethylene, delays fruit ripening via the inhibition of ethylene receptor. However, unsuitable 1-MCP treatment can cause fruit ripening disorders.

**Results:**

In this study, we show that short-term 1-MCP treatment (400 nL•L^− 1^, 2 h) significantly delays papaya fruit ripening with normal ripening characteristics. However, long-term 1-MCP treatment (400 nL•L^− 1^, 16 h) causes a “rubbery” texture of fruit. The comparative transcriptome analysis showed that a total of 5529 genes were differently expressed during fruit ripening compared to freshly harvested fruits. Comprehensive functional enrichment analysis showed that the metabolic pathways of carbon metabolism, plant hormone signal transduction, biosynthesis of amino acids, and starch and sucrose metabolism are involved in fruit ripening. 1-MCP treatment significantly affected fruit transcript levels. A total of 3595 and 5998 differently expressed genes (DEGs) were identified between short-term 1-MCP, long-term 1-MCP treatment and the control, respectively. DEGs are mostly enriched in the similar pathway involved in fruit ripening. A large number of DEGs were also identified between long-term and short-term 1-MCP treatment, with most of the DEGs being enriched in carbon metabolism, starch and sucrose metabolism, plant hormone signal transduction, and biosynthesis of amino acids. The 1-MCP treatments accelerated the lignin accumulation and delayed cellulose degradation during fruit ripening. Considering the rubbery phenotype, we inferred that the cell wall metabolism and hormone signal pathways are closely related to papaya fruit ripening disorder. The RNA-Seq output was confirmed using RT-qPCR by 28 selected genes that were involved in cell wall metabolism and hormone signal pathways.

**Conclusions:**

These results showed that long-term 1-MCP treatment severely inhibited ethylene signaling and the cell wall metabolism pathways, which may result in the failure of cell wall degradation and fruit softening. Our results reveal multiple ripening-associated events during papaya fruit ripening and provide a foundation for understanding the molecular mechanisms underlying 1-MCP treatment on fruit ripening and the regulatory networks.

**Electronic supplementary material:**

The online version of this article (10.1186/s12870-019-1904-x) contains supplementary material, which is available to authorized users.

## Background

Papaya fruit is a popular fruit known for its sweet and exotic flavor and nutritional value [1]. However, papaya is highly perishable, ripens, and rapidly deteriorates after harvest, thus restricting its market promotion [2]. As a typical climacteric fruit, the ripening of papaya is determined by ethylene [3, 4]. 1-MCP has also been applied to maintain papaya fruit quality and extend its shelf life [5, 6]. However, inappropriate 1-MCP treatment tends to cause an elastic state or “rubbery” texture in papaya [5, 7, 8]. A few studies have focused on these issues, and the related mechanism of this phenomenon has not been thoroughly investigated.

Ethylene is an important plant hormone that regulates plant growth and developmental processes, including ripening and senescence, and it also profoundly affects the quality of harvested products [9]. Ethylene is known to trigger ripening in climacteric fruits and senescence in non-climacteric fruits, vegetables, and ornamental plants [3]. Manipulating ethylene production is an effective way to either promote rapid and predictable ripening of climacteric fruits or to delay ripening.

The postharvest technologies of controlling ethylene and the ethylene pathway have been extensively studied [5, 10, 11] .1-Methylcyclopropene (1-MCP) is an ethylene receptor inhibitor and a non-toxic antagonist of ethylene that has been employed to increase the shelf life of various climacteric and non-climacteric fruits by effectively delaying fruit ripening and softening [5, 12–17]. By binding to ethylene receptors, 1-MCP acts as an efficient antagonist and exerts a persistent effect. Fruit producers have applied 1-MCP as a ripening delayer to prevent non-homogeneous ripening or sudden softening of fruits caused by exposure to exogenous ethylene or poor postharvest handling [6]. The broad application of 1-MCP advances commercial agriculture, as well as improves our understanding and provides insights into the mechanisms underlying plant ethylene responses. However, several problems and questions relating to the practical application of 1-MCP remain [14, 18]. For example, 1-MCP treatment inhibits the production of many volatile alcohols and esters [19] and causes core browning [20] in apple and softening disorder in papaya fruit [5]. Unsuitable 1-MCP treatment (long duration, high treated concentration or low fruit maturity) may cause banana to stay green or ripen with uneven color [14].

RNA sequencing (RNA-Seq) analysis is a powerful tool commonly used to study transcriptomes [21, 22]. The present study aimed to explore the global view of transcript level of papaya fruit treated with or without 1-MCP treatment using RNA-Seq technique. Suitable and unsuitable 1-MCP treatments were conducted to study the transcriptomic differences between normal-ripening fruits and fruits with ripening disorder to identify key factors involved in papaya fruit ripening and ripening disorder caused by 1-MCP treatment.

## Results

### Physiological characterization during fruit ripening under different 1-MCP treatments

**Fig. 1:**
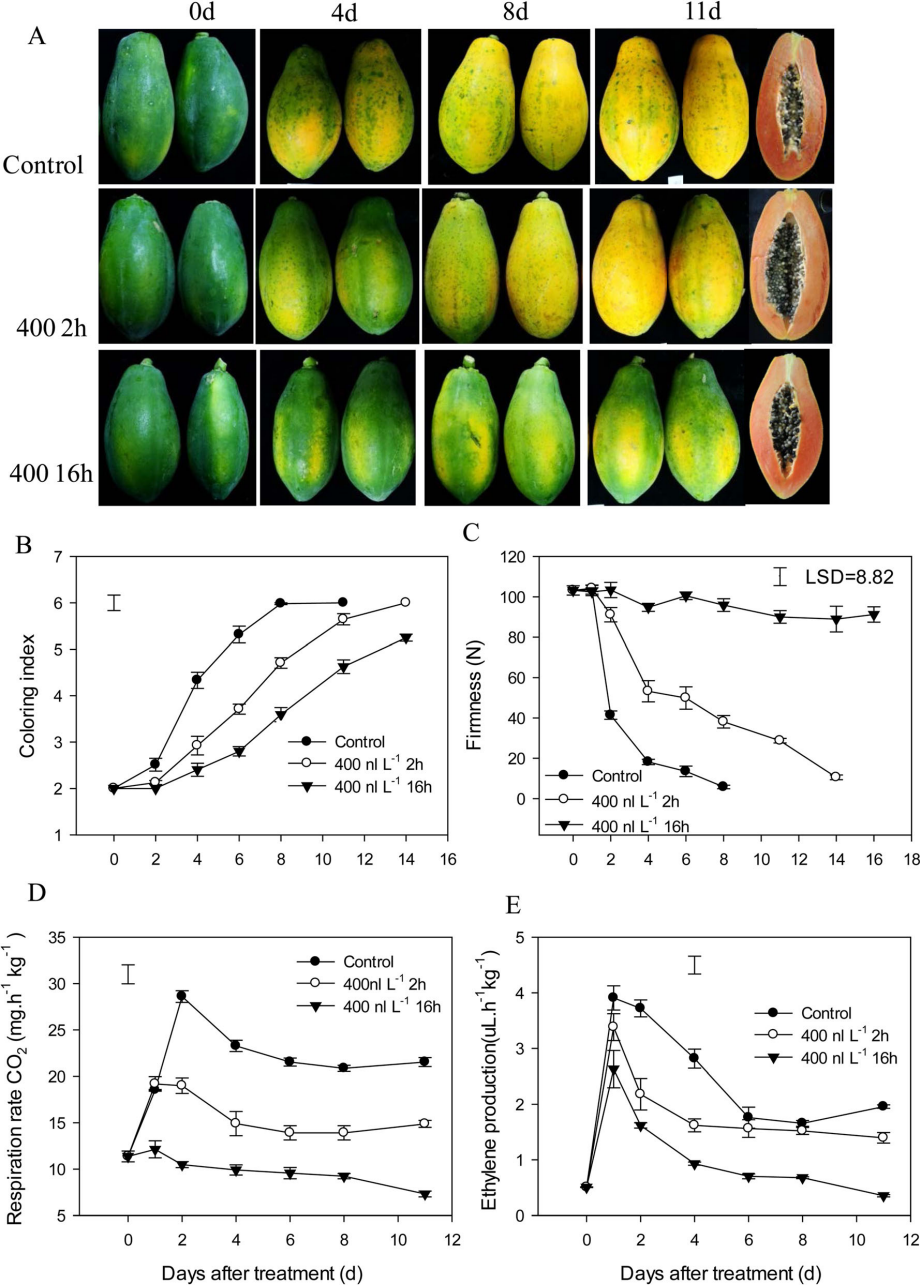
Effects of 1-MCP treatment on the firmness, coloring index, respiration rate, and ethylene production of papaya fruit. **a**, Pictures of papaya fruit during the storage with 1-MCP treatments. **b**, Changes in coloring index, **c**, fruit firmness, **d** and **e**, fruit respiration rate and ethylene production. Control, fruit directly treated with ethephon for ripening; 400 2 h, fruit treated with 400 nL•L^− 1^ of 1-MCP for 2 h followed by ethephon treatment; 400 16 h, fruit treated with 400 nL•L^− 1^ of 1-MCP for 16 h followed by ethephon treatment. Three biological replicates were analyzed and vertical bars indicate the SE. Least significant difference (LSD) at *P* = 0.05 was calculated to compare the differences between means

Figure 1 shows that 1-MCP treatment delayed fruit ripening, including fruit coloring and softening (Fig. 1a–c), especially for the long-term 1-MCP treatment (400 nL•L^− 1^,16 h). Fruit ripening rapidly occurred in the control group. Fruit coloring index rapidly increased from second day after treatment in the control group. Both 1-MCP treatments delayed fruit coloring, which turned completely yellow during storage (Fig. 1a and b). In terms of fruit firmness, the fruits in the control group rapidly softened on the second day and declined to low level of 10 N on the sixth day. Short-term 1-MCP treatment effectively delayed the decrease in firmness, whereas fruits treated with long-term 1-MCP treatment remained firm during the entire storage period (Fig. 1a and c). Both 1-MCP treatments significantly reduced peak fruit respiration rates, and long-term 1-MCP treatment severely inhibited fruit respiration (Fig. 1 d). For ethylene production, both 1-MCP treatments decreased the ethylene peak and reduced ethylene production during the later storage period (Fig. 1e). Long-term 1-MCP treatment more extensively repressed fruit respiration and ethylene production. In general, while both 1-MCP treatments delayed fruit ripening, long-term 1-MCP treatment led to the “rubbery” fruit ripening disorder texture.

### 1-MCP treatments affect fruit cell structure during fruit ripening

**Fig. 2:**
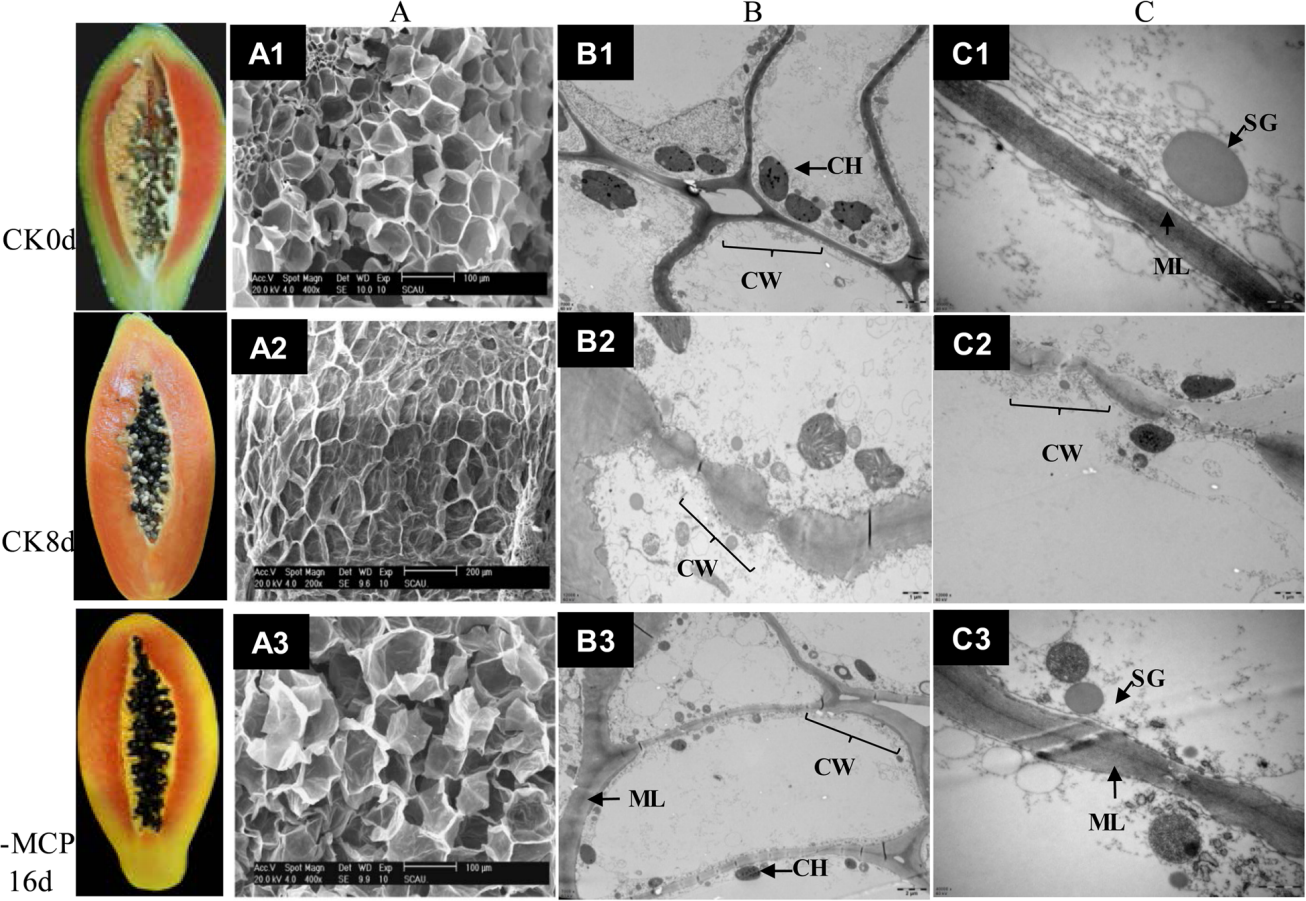
Microstructure observations of the fruit cell walls after 1-MCP treatment by scanning electron microscopy (SEM) (**a**) and transmission electron microscopy (TEM) (**b**, **c**). Fruit pulp samples from the control fruit of 0 days and 8 days after treatment, and fruit with 400 nL·L^− 1^ 1-MCP treatment of 16 days after treatment are presented. **a**, Fruit cell wall microstructure observed by SEM. The scale bar is 100 μm. **b**, **c**, Fruit cell wall ultrastructure observed by TEM. The scale bar is 2 μm. CW, cell wall; PM, plasma membrane; ML, intercellular layer; SG, starch granules; MF, microfiber; M, mitochondrion; CH, chloroplast

Fruit cell structure was intact, and cell outline was clear in the freshly harvested papaya fruit (Fig. 2a1). The individual cells with a clear cellular framework were arranged in an orderly and tight manner in the fruits that were just harvested (Fig. 2a1), where this result also presented in the long-term 1-MCP-treated fruits (Fig. 2a3). The cell wall shrank and degraded and was thinner and obscure on the eighth day of storage (Fig. 2a2). The cell wall ultrastructure observed by TEM showed similar results with SEM. Fruits showed a complete cell wall (CW) structure just after harvest on 0 day (Fig. 2b1 and c1), which showed intact and clear cell organelle [microfibrous filaments (MF), CH (chloroplast), SG (starch granules)]. Cell wall composition degraded completely and a large amount of flocculent substance was observed in the cytoplasm. The completed organelle structure could not be found at this stage (Fig. 2b2 and c2). The cell wall structure was still intact in 1-MCP-treated fruit at the 16th day (Fig. 2b3 and c3), partial degradation of the outer cell walls were observed, and organelles not clear as the fresh harvested, but they were much better than the control; the cell structure was still complete and clear. Some organelles still could be observed (Fig. 2c13).

### RNA-Seq analyses for the transcriptome of fruit under different 1-MCP treatments

A total of 144.35 gigabytes (Gb) of clean reads from the samples were obtained following quality assessment and data filtering. The average clear data for each sample was about 5.28 Gb. The GC% of sequenced data from 21 libraries ranged from 44.55 to 46.05%, and the percentage of reads with an average quality score > 30 was about 91% (Additional file 6: Table S2), indicating that the accuracy and quality of the sequencing data are sufficient for further analysis. The general sequencing statistics are shown in Additional file 6: Table S2. The mapping efficiency of 21 samples to the papaya (*C. papaya*) genome range from 69.23–78.77%, as shown in Additional file 6: Table S2. Only uniquely mapped reads were used in the subsequent analysis of gene expression profiles in different treatment groups. The correlation coefficient analysis between each pair of three biological replicates showed that the estimated gene expression levels of any replicate pair of each treatment were highly consistent with each other (Additional file 1: Figure S1a), and the replicates showed consistent expression level with each other (Additional file 1: Figure S1b). However, different samples using various treatments were differentially expressed.

A total of 23,712 transcripts were identified from all of the samples based on the blast, which accounted for 84.9% of the annotated genes in papaya (Additional file 10: Data S1). In addition, 1098 new genes were identified, of which, 778 were annotated using BLAST. Approximately 27.17% of the genes showed very strong homology (80–100%) to the gene sequences in the database and the other 45.3% showed 60–80% identity (Additional file 2: Figure S2a).

A total of 15,032 genes annotated in the GO database were categorized into 50 functional groups, belonging to three main GO ontologies: biological processes, cellular components, and molecular functions (Additional file 1: Figure S1c). The ‘metabolic process’ (9895 genes, 65.82%), cellular process (8513 genes, 56.63%), and catalytic activity (7630 genes, 50.75%) were the three predominant classifications among the functional groups.

For COG analysis, 7112 putative proteins were clustered into 25 functional categories. Among these, ‘general function prediction only’ (2094, 19.84%) accounted for the largest fraction, followed by ‘transcription’ (10.23%) and ‘replication, recombination and repair’ (9.8%). In addition, 8.61% of assembled genes were assigned to signal transduction mechanisms. The ‘cell wall/membrane/envelope biogenesis’ category accounted for 2.39% (Additional file 2: Figure S2b).

### Transcriptomic analysis during fruit ripening

**Fig. 3:**
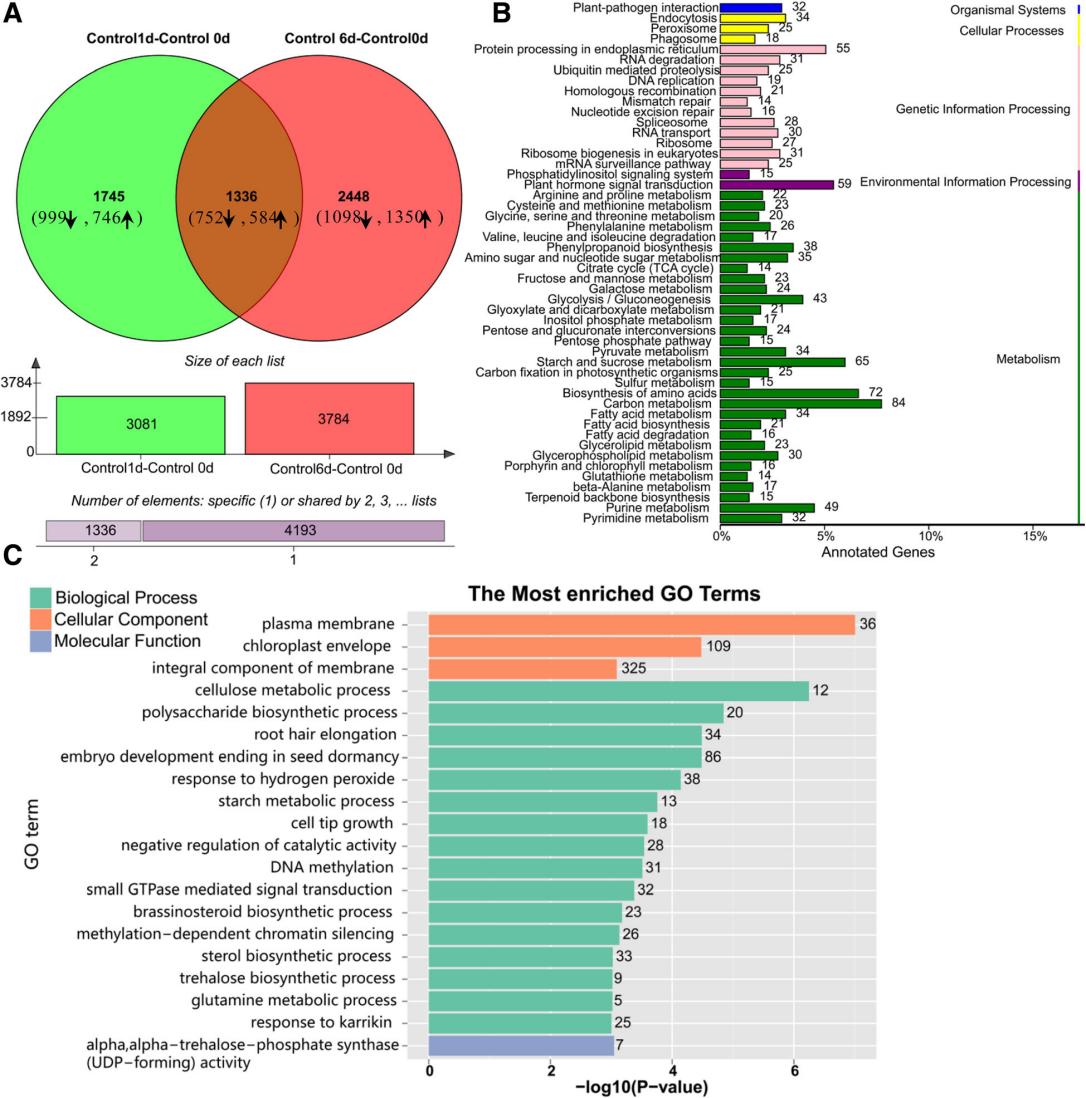
Differentially expressed genes (DEGs) involved in the fruit ripening process. **a**, Venn diagrams showing the overlap of the DEGs (| FC| ≥ 2) that were upregulated (UP) and downregulated (DW) in the papaya fruit at 1 and 6 days after treatment (DAT) compared to 0 DAT under the control condition. The arrow up and down directions indicated the number of the up- and downregulated genes. Software (http://bioinformatics.psb.ugent.be/webtools/Venn/) was used for the Venn diagram. **b**, **c** Histogram of GO term and KEGG pathway classification of DEGs in the control condition. **b**, Most enriched pathways identified with KEGG Orthology-Based Annotation System (KOBAS) 2.0 in papaya fruit after they were harvested at 1 d and 6 d compared to 0DPT. **c**, Top 20 GO terms identified from sample under control condition in 1 DAT vs 0 DAT and 6 DATvs 0 DAT

A total of 5529 genes were differently expressed (FC ≥ two-fold) during fruit ripening process compared to fruit just harvested (1 DAT(days after treatment) vs. 0 DAT and 6 DAT vs. 0 DAT) (Fig. 3a), of these differently expressed genes (DEGs), 3081 were at 1 DAT, 3784 were at 6 DAT, and 1336 DEGs were identified at both stages (Fig. 3a). The number of DEGs increased following fruit ripening (Fig. 3a).

KEGG analysis assigned the DEGs to 125 metabolic pathways (each of which contained one or more DEGs) (Additional file 11: Data S2). Figure 3b shows the 52 most enriched metabolic/biological pathways. Notably, carbon metabolism, biosynthesis of amino acids, starch and sucrose metabolism, and plant hormone signal transduction were the most enriched pathways, in which 84, 72, 65, and 59 DEGs were identified, respectively. These results showed that the metabolic pathways of carbon metabolism, plant hormone signal transduction, biosynthesis of amino acids, and starch and sucrose metabolism are important to fruit ripening (Fig. 3b).

The 20 most enriched GO terms are presented in Fig. 3c. The cellular component of integral component of membrane, chloroplast envelope, and plasma membrane were the most enriched categories, including 325, 109, and 36 DEGs, respectively (Fig. 3c). In terms of biological process, embryo development ending in seed dormancy, response to hydrogen peroxide, DNA methylation, sterol biosynthetic process, and small GTPase-mediated signal transduction are the most enriched categories. The UDP-forming activity was the only molecular function categories in the top 20 enriched GO terms. More biological process categories were enriched than cellular component and molecular function.

Around 73 genes were found to be involved in hormone signal pathway, of which, 18, 36, 9, and 10 genes involved in the ethylene signal pathway, auxin signal pathway, abscisic acid (ABA) pathway, and gibberellin (GA) pathway, respectively (Additional file 7: Table S3). Some genes were selected (log _2_ | FC| ≥ 2) and presented in Table 1. Several important regulators involved in ethylene synthesis and signal transduction were identified, including *ERFs* (Ethylene-responsive transcription factor) (7), *CTR1*(1), *ACO (*ACC oxidase*)* (1), and *ERS (*ethylene response sensor*)* (1) (Table 1). The expression levels of three *ERF*s were significantly repressed during fruit ripening, and those of the other four *ERF*s were dramatically increased during fruit ripening, especially for *ERF-WRI1*, which reached more than [500-fold] on the sixth day compared to 0 DAT (Table 1). The transcript levels of *ACO* and *CTR1* genes were enhanced with the fruit ripening process. More DEGs involved in auxin transport, synthesis, and signal transduction were identified during fruit ripening (Table 1). Most auxin transport genes were downregulated during fruit ripening, whereas auxin responsive genes, such as *ARF*s (Auxin response factors) and *IAA*s (indoleacetic acid), were upregulated during fruit ripening (Table 1). The three ABA receptor genes were significantly upregulated with fruit ripening. Genes involved in the GA pathway were also significantly differentially expressed during fruit ripening (Table 1 and Additional file 7: Table S3).

**Table 1:** Selected DEGs involved in hormone signal pathway

Gene ID	log_2_ FC		Blast annotation	Blasted species
	DAT1/0	DAT6/0		
Ethylene				
evm.TU.supercontig_62.11	− 5.61	− 4.60	Ethylene-responsive transcription factor (ERF109)	*Arabidopsis thaliana*
evm.TU.supercontig_2304.1	−3.17	1.03	Ethylene-responsive transcription factor (ERF)	*Arabidopsis thaliana*
evm.TU.supercontig_224.4	−2.90	− 1.17	AP2-like ethylene-responsive transcription factor (ERF)	*Arabidopsis thaliana*
evm.TU.supercontig_151.32	2.06	−0.26	Ethylene response sensor 1 (ERS1)	*Arabidopsis thaliana*
evm.TU.supercontig_152.58	2.25	1.86	1-aminocyclopropane-1-carboxylate oxidase 1 (ACO1)	*Petunia hybrida*
evm.TU.supercontig_34.132	2.60	2.21	Ethylene-responsive transcription factor (ERF053)	*Arabidopsis thaliana*
evm.TU.supercontig_128.50	2.78	2.18	Serine/threonine-protein kinase (CTR1)	*Arabidopsis thaliana*
evm.TU.supercontig_199.10	3.41	2.43	AP2-like ethylene-responsive transcription factor(ERF)	*Arabidopsis thaliana*
evm.TU.supercontig_49.94	4.68	2.23	Ethylene-responsive transcription factor (ERF110)	*Arabidopsis thaliana*
evm.TU.supercontig_54.28	5.90	9.33	Ethylene-responsive transcription factor WRI1(ERF-WRI1)	*Arabidopsis thaliana*
Auxin				
evm.TU.supercontig_26.24	−4.54	−3.50	Auxin response factor 5 (ARF5)	*Arabidopsis thaliana*
evm.TU.supercontig_3603.1	−4.16	−2.15	Auxin transport protein BIG (BIG)	*Arabidopsis thaliana*
evm.TU.contig_35483	−4.13	−0.56	Auxin efflux carrier component 1 (PIN1)	*Arabidopsis thaliana*
evm.TU.contig_44255	−3.63	0.06	Auxin efflux carrier component 1 (PIN1)	*Arabidopsis thaliana*
evm.TU.supercontig_87.29	−2.59	0.40	Auxin-responsive protein IAA11(IAA11)	*Arabidopsis thaliana*
evm.TU.supercontig_327.9	−2.39	−1.52	Auxin-repressed 12.5 kDa protein	*Fragaria ananassa* (Strawberry)
C.papaya_newGene_850	−2.23	−1.76	Auxin transport protein BIG (BIG)	*Arabidopsis thaliana*
evm.TU.supercontig_34.122	1.97	−2.16	Probable indole-3-acetic acid-amido synthetase (GH3.5)	*Oryza sativa subsp. japonica* (Rice)
evm.TU.supercontig_6.357	2.06	1.16	Auxin efflux carrier family protein isoform 1	*Theobroma cacao*
evm.TU.supercontig_395.4	2.06	0.86	Cytochrome b561 and DOMON domain-containing protein	*Arabidopsis thaliana*
evm.TU.contig_31756	2.12	1.05	Auxin response factor 2 (ARF2)	*Arabidopsis thaliana*
evm.TU.supercontig_37.56	2.35	0.14	Auxin-induced protein X10A	*Glycine max* (Soybean)
evm.TU.contig_38023	2.71	2.08	Auxin response factor 2 GN = MTG10.3 (ARF2)	*Arabidopsis thaliana* (Mouse-ear cress)
evm.TU.supercontig_9.240	3.32	1.84	Auxin-regulated gene involved in organ size	*Theobroma cacao*
evm.TU.supercontig_34.167	3.68	1.93	Auxin-induced protein 6B	*Glycine max* (Soybean)
evm.TU.supercontig_23.159	4.23	1.27	Auxin-induced protein 22B AUX22B	*Vigna radiata var. radiata* (Mung bean)
evm.TU.supercontig_292.1	4.30	3.60	Probable indole-3-acetic acid-amido synthetase (GH3.1)	*Arabidopsis thaliana*
evm.TU.supercontig_150.1	4.37	3.87	Indole-3-acetic acid-induced protein ARG7	*Vigna radiata* var. radiata (Mung bean)
evm.TU.supercontig_26.221	5.52	–	Indole-3-acetic acid-induced protein ARG7	*Vigna radiata* var. radiata (Mung bean)
evm.TU.supercontig_37.221	5.78	–	Auxin-induced protein X10A	*Glycine max* (Soybean)
gevm.TU.contig_24967.2	8.11	4.92	Auxin efflux carrier family protein, putative	*Theobroma cacao*
evm.TU.supercontig_3.174	–	7.43	WAT1-related protein	*Arabidopsis thaliana*
Abscisic acid				
evm.TU.supercontig_19.124	1.15	2.04	Abscisic acid receptor PYL4	*Arabidopsis thaliana*
evm.TU.contig_36665	2.12	0.85	Abscisic acid receptor PYR1	*Arabidopsis thaliana*
evm.TU.supercontig_3.59	–	2.76	Abscisic acid receptor PYL8	*Arabidopsis thaliana*
Gibberellin				
evm.TU.supercontig_26.40	−2.73	−3.34	Gibberellin regulated protein	
evm.TU.supercontig_66.47	−2.11	−4.76	Gibberellin-regulated protein 3 (GASA3)	*Arabidopsis thaliana*
evm.TU.supercontig_166.2	−1.88	−5.25	Gibberellin 2-beta-dioxygenase (GA2OX1)	*Phaseolus coccineus* (Scarlet runner bean)
evm.TU.supercontig_18.158	1.69	2.88	Gibberellin receptor GID1, putative	*Theobroma cacao*
evm.TU.supercontig_3097.1	2.58	2.01	GRAS domain family	
evm.TU.supercontig_77.94	5.12	2.22	Gibberellin 2-beta-dioxygenase 1	*Pisum sativum* (Garden pea)

More than 136 DEGs are involved in cell-wall metabolism (Additional file 8: Table S4), including cellulose synthesis, sucrose synthase, pectin metabolism (*PME, PG, PE, GAUT*), hemicellulose metabolism (*EXP, EXY1, β-GAL, BGL, EGase*), and lignin metabolism (*PAL, 4CL, CCR, POD*). Some selected important DEGs (log _2_ | FC| ≥ 2) involved in cell-wall metabolism are listed in Table 2. Several of these genes were repressed with fruit ripening, whereas most of them dramatically increased during fruit ripening. The *EXY1* gene was upregulated by more than [2000-fold] on the first day of ethephon treatment compared to 0 DAT, and *PG* gene was upregulated by more than [250-fold], too (Table 2). The expression levels of other genes, such as *Pectinesterase, XTH1,* and *CS,* were also significantly upregulated during fruit ripening (Table 2 Additional file 8: Table S4). These results coincided with the fruit softening phenotype.

**Table 2:** Selected DEGs involved in cell wall metabolism pathway

Gene ID	log_2_ FC		Blast annotation	Blasted species
	DAT1/0	DAT6/0		
evm.TU.contig_39949	−4.26	−3.48	Tubulin beta-6 chain MXC9.21	*Arabidopsis thaliana*
evm.TU.supercontig_3.77	−4.17	−4.16	Pectinesterase 34 (PE34)	*Arabidopsis thaliana*
evm.TU.supercontig_40.35	−3.73	−1.70	Probable glycosyltransferase	*Arabidopsis thaliana*
evm.TU.supercontig_69.40	−3.67	−1.37	Probable polygalacturonase non-catalytic subunit JP650 (PG)	*Arabidopsis thaliana*
evm.TU.supercontig_50.101	−3.33	0.51	Callose synthase 7	*Arabidopsis thaliana*
evm.TU.supercontig_5.99	−3.23	1.05	Probable 3-deoxy-D-manno-octulosonic acid transferase (KDO transferase)	*Arabidopsis thaliana*
evm.TU.supercontig_5185.1	−3.12	−4.05	Cellulose synthase A catalytic subunit 6 [UDP-forming]	*Arabidopsis thaliana*
evm.TU.supercontig_145.14	−3.06	−3.89	Pectinesterase 36 (Precursor) (PE36)	*Arabidopsis thaliana*
evm.TU.supercontig_69.14	−3.02	−4.14	Probable xyloglucan endotransglucosylase/hydrolase protein 33 (XTH33)	*Arabidopsis thaliana*
evm.TU.supercontig_233.22	−2.91	−0.53	Probable xyloglucan endotransglucosylase/hydrolase protein 25 (XTH25)	*Arabidopsis thaliana*
evm.TU.supercontig_452.5	−2.84	−1.42	Callose synthase 3	*Arabidopsis thaliana*
evm.TU.supercontig_714.1	−2.70	−0.99	Putative callose synthase 8	*Arabidopsis thaliana*
evm.TU.supercontig_25.167	−2.69	−0.24	Callose synthase 3	*Arabidopsis thaliana*
evm.TU.supercontig_260.6	−2.59	0.25	Expansin-A4 (EXP-A4)	*Arabidopsis thaliana*
evm.TU.supercontig_341.1	−2.52	−2.18	Galactoside 2-alpha-L-fucosyltransferase (FUT2)	*Pisum sativum*
evm.TU.supercontig_157.42	−2.33	−1.54	Xyloglucan galactosyltransferase (MUR3)	*Arabidopsis thaliana*
evm.TU.supercontig_49.102	−2.22	−2.53	Probable cellulose synthase A catalytic subunit 9 (CS)	*Arabidopsis thaliana*
evm.TU.supercontig_180.28	−2.09	−1.09	Probable polygalacturonase (PG)	*Vitis vinifera* (Grape)
evm.TU.supercontig_217.19	−2.08	−0.20	Probable sucrose-phosphate synthase 1(SPS1)	*Citrus unshiu* (Satsuma mandarin)
evm.TU.supercontig_79.25	2.03	2.04	UDP-glucose 4-epimerase (GAE)	*Cyamopsis tetragonoloba*
evm.TU.supercontig_1145.1	2.28	0.47	Cellulose synthase-like protein D3 (CS-D3)	*Arabidopsis thaliana*
evm.TU.supercontig_1.107	2.29	0.05	UDP-glucose 6-dehydrogenase 2 (UGDH2)	*Arabidopsis thaliana*
evm.TU.supercontig_92.75	2.35	0.78	Tubulin beta-3 chain	*Gossypium hirsutum*
evm.TU.supercontig_64.43	2.51	0.62	Probable galacturonosyltransferase-like 1 (GalAT-like 1)	*Arabidopsis thaliana*
evm.TU.supercontig_198.12	2.51	−1.48	Probable xyloglucan endotransglucosylase/hydrolase protein 8 (XTH8)	*Arabidopsis thaliana*
evm.TU.supercontig_37.20	2.52	4.83	Beta-galactosidase 10 (β-GAL10)	*Arabidopsis thaliana*
evm.TU.supercontig_1.388	2.56	4.10	UDP-glucuronic acid decarboxylase 6 (UGDL6)	*Arabidopsis thaliana*
evm.TU.supercontig_285.6	2.63	1.52	UDP-arabinose 4-epimerase 1	*Arabidopsis thaliana*
evm.TU.supercontig_1.419	3.09	1.00	Expansin-A4 (EXP-A4)	*Arabidopsis thaliana*
evm.TU.supercontig_808.2	3.12	−0.12	Glucuronoxylan 4-O-methyltransferase 1	*Arabidopsis thaliana*
evm.TU.supercontig_146.68	3.13	4.74	Galactinol synthase 2 (GS2)	*Arabidopsis thaliana*
evm.TU.supercontig_208.1	3.47	1.81	Probable xyloglucan endotransglucosylase/hydrolase protein 30 (XTH30)	*Arabidopsis thaliana*
evm.TU.supercontig_1.407	3.51	1.20	Glucan endo-1,3-beta-glucosidase	*Triticum aestivum* (Wheat)
evm.TU.supercontig_18.107	3.84	2.61	UDP-glucuronate 4-epimerase 3 (GAE3)	*Arabidopsis thaliana*
evm.TU.supercontig_151.19	4.09	2.85	Pectinesterase 40 (PE40)	*Arabidopsis thaliana*
evm.TU.supercontig_6.101	4.42	4.45	Microtubule-associated protein RP/EB family member 1C (MAPRE1C)	*Arabidopsis thaliana*
evm.TU.supercontig_92.36	4.52	6.16	Polygalacturonase (PME)	*Actinidia deliciosa*
evm.TU.supercontig_189.36	4.79	5.85	Beta-glucosidase 32 (β-GAL32)	*Arabidopsis thaliana*
evm.TU.supercontig_57.23	5.35	–	Chitinase 10 (Precursor)	*Oryza sativa* subsp. japonica
evm.TU.supercontig_2.231	5.45	6.57	Endoglucanase 8 (Precursor) (Egase8)	*Arabidopsis thaliana*
evm.TU.supercontig_46.178	6.52	–	Cellulose synthase A catalytic subunit 7 (CS-A7)	*Arabidopsis thaliana*
evm.TU.supercontig_46.179	6.77	1.17	Cellulose synthase A catalytic subunit 7 (CS-A7)	*Arabidopsis thaliana*
evm.TU.supercontig_250.6	8.28	7.61	Polygalacturonase (PG)	*Prunus persica* (Peach)
evm.TU.supercontig_64.7	8.42	–	Polygalacturonase (PG)	*Prunus persica* (Peach)
evm.TU.supercontig_106.45	11.59	10.04	Endoxylanase (EXY1)	*Carica papaya*
evm.TU.supercontig_1540.1	–	5.43	Pectinesterase 12 (PE12)	*Arabidopsis thaliana*
evm.TU.supercontig_33.138	–	3.56	Pectinesterase 46 (PE46)	*Arabidopsis thaliana*
evm.TU.supercontig_50.160	–	5.14	Sucrose synthase 2 (SS2)	*Arabidopsis thaliana*
evm.TU.supercontig_8.136	–	5.92	Putative xyloglucan endotransglucosylase/hydrolase protein 1 (XTH1)	*Arabidopsis thaliana*
evm.TU.supercontig_81.134	–	2.78	Expansin-A4 (EXP-A4)	*Arabidopsis thaliana*

### Transcriptomic analysis after 1-MCP treatment

**Fig. 4:**
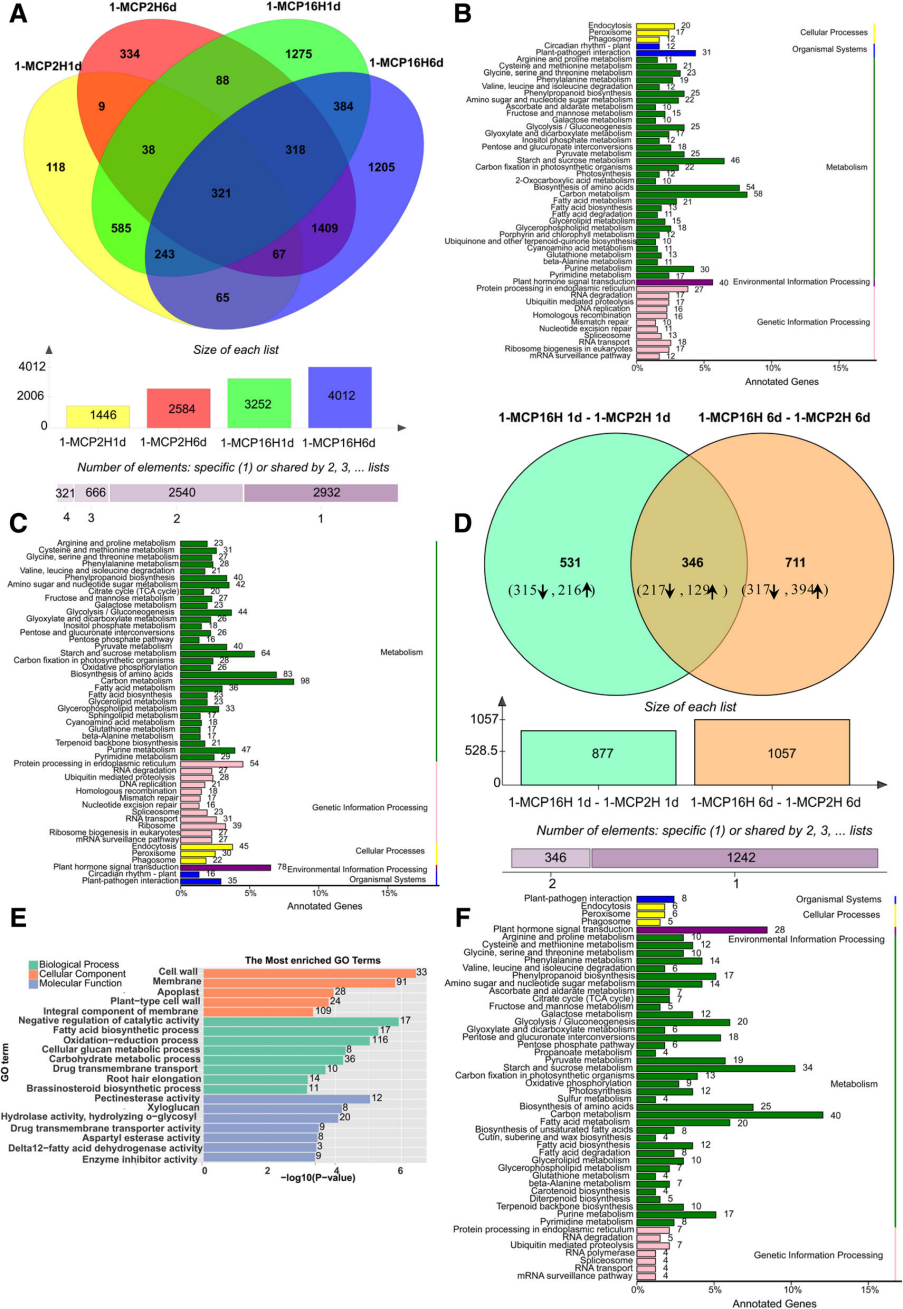
Effect of 1-MCP treatments on the transcripmics analysis. **a**, The number of DEGs derived from comparison between the 1-MCP (2 h) on 1 d and 6 d samples and the control samples at each time point and the number of DEGs derived from comparison between the 1-MCP (16 h) on 1 d and 6 d samples and the control samples at each time point. **b**, The number of DEGs derived from comparison between the 1-MCP (16 h) on 1 d and 6 d samples and the 1-MCP (2 h) samples at each time point. Up and down arrows denote genes up- or down-regulated treated samples. Software (http://bioinformatics.psb.ugent.be/webtools/Venn/) was used for the Venn diagram. **c**, **d**, **e** The most enriched pathways identified with KEGG Orthology-Based Annotation System (KOBAS) 2.0 in papaya fruit after they were harvested at 1 d and 6 d under 1-MCP treatment of 2 h and 16 h compared to control (**c**, **d**), and in comparison of 1-MCP treatment (400, 16 h) and the 1-MCP (400, 2 h) condition on 1DAT and 6 DAT (**e**), respectively. **f**, Top 20 enrichment GO term of DEGs comparison of 1-MCP treatment (400, 16 h) and the 1-MCP (400, 2 h) condition on 1 DAT and 6 DAT

Figure 1 shows that 1-MCP treatments significantly delayed or inhibited fruit softening and ripening. To obtain a global overview of the genes involved in 1-MCP response, we analyzed the transcriptomes between control group and 1-MCP treatments. Figure 4a shows that a total of 6459 genes were differently expressed (≥ 2-fold) between 1-MCP treatments (both long-term and short-term 1-MCP treatments) and the control. The number of DEGs increased with storage time and duration of 1-MCP treatment (Fig. 4a). 2932 DEGs were identified specifically in each group, and 321 DEGs were identified in all of the groups. A total of 3595 genes were differently expressed between short-term 1-MCP treatment and the control (Fig. 4a), of which 1446 were at 1 DAT, 2584 were at 6 DAT, and 435 were shared at both time points. A higher number of DEGs were identified at 6 DAT than 1 DAT. More DEGs were upregulated than downregulated at 1DAT. However, more DEGs were downregulated by 1-MCP at 6 DAT (Additional file 3: Figure S3a). A higher number of DEGs were identified under long-term 1-MCP treatment, with a total of 5998 genes being differently expressed between long-term 1-MCP treatment and the control (Fig. 4a and. Additional file 3: Figure S3b). These results indicated that 1-MCP significantly affects fruit transcript levels, and long-term 1-MCP treatment induces more severe effects.

For GO analysis, cellulose metabolic process, carotenoid biosynthetic process, and chloroplast envelope were the most significantly enriched terms in the short-term 1-MCP vs. the control group (Additional file 3: Figure S3c). For the comparison of long-term 1MCP vs. control group, plasma membrane, cellulose metabolic process, root hair elongation, and fatty acid biosynthetic process were the most significantly enriched terms (Additional file 3: Figure S3d). Figure 4b-c identify the 50 most enriched metabolic/biological pathways for the comparison of the short-term 1-MCP vs. control and long-term 1MCP vs. control group, respectively. Carbon metabolism, biosynthesis of amino acids, starch and sucrose metabolism, and plant hormone signal transduction were the most enriched pathways in both comparison (Fig. 4b,c). However, much more DEGs were enriched in each pathway for the comparison of the long-term 1MCP vs. control group,. A higher number of DEGs were also enriched in other pathways for the long-term 1-MCP treatment than the short-term 1-MCP treatment.

When long-term 1-MCP treatment was compared to short-term 1-MCP treatment, a total of 1934 DEGs were identified, which may play a role in the fruit ripening disorder (Fig. 4d). Among which, 877 DEGs were at 1 DAT and 1057 were at 6 DAT, and 346 DEGs were shared at both time points. GO categories analysis showed that the most significantly enriched terms were cell wall, membrane, negative regulation of catalytic activity, fatty acid biosynthetic process, oxidation-reduction process, pectinesterase activity, and xyloglucan (*P* < 0.0001) (Fig. 4e). The GO terms related to cell wall metabolism were most significantly enriched.

KEGG analysis showed that the most enriched pathways were carbon metabolism, starch and sucrose metabolism, plant hormone signal transduction, and biosynthesis of amino acids and glycolysis (Fig. 4f and Additional file 4: Figure S4). These results showed that the cell wall metabolism pathway and the plant hormone signal transduction pathways were most enriched, indicating that the cell wall metabolism pathway and hormone signal pathways are closely related to papaya fruit ripening disorder.

### Candidate DEGs involved in fruit ripening

**Fig. 5:**
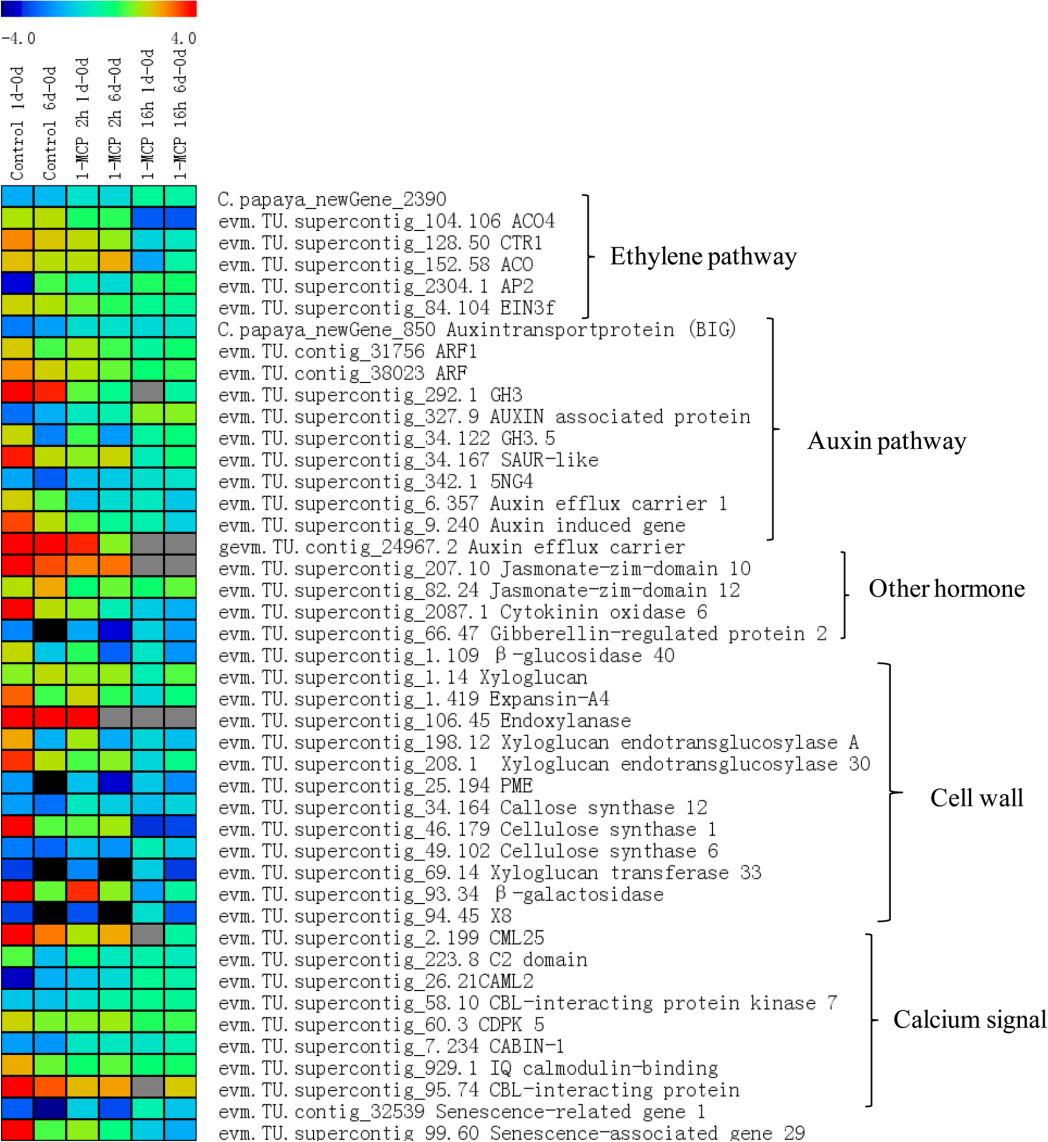
Heat map showing the expression levels of several selected genes associated with the hormone signal, cell wall metabolic, and calcium signal pathways between short-term 1-MCP, long-term 1-MCP, and control group. at 1 and 6 DAT compared to 0 DAT

Figure 5 shows several selected DEGs involved in the hormone signal pathway, cell wall metabolism, and calcium signaling, which were significantly increased during the fruit ripening process. However, the expression levels of most of these genes were repressed by 1-MCP treatments (Fig. 5). For example, genes involved in ethylene synthesis *ACO* and signal transduction *CTR1* and *EIN3f* were significantly upregulated during fruit ripening, but 1-MCP treatments dramatically repressed their expression. The transcript levels of *GH3, SAUR,* and *Auxin efflux carrier* involved in the auxin signal pathway increased with fruit ripening, but dramatically repressed by 1-MCP treatments. Other hormone signal components involved in the jasmonic acid (JA), cytokinin (CK), and GA pathways were also reduced by 1-MCP treatments (Additional file 9: Table S5). Notably, genes involved in cell wall metabolism dramatically increased with fruit ripening, such as *endoxylanase (EXY1), CS,* and *Xyloglucan endotransglucosylase 30*, but were severely repressed by 1-MCP treatments. Calcium signal also involved in fruit ripening, such as *CML25* and *CBL-interacting protein*, increased with fruit ripening. The 1-MCP treatments repressed their expression during fruit ripening.

### DEGs related to cell wall metabolism

**Fig. 6:**
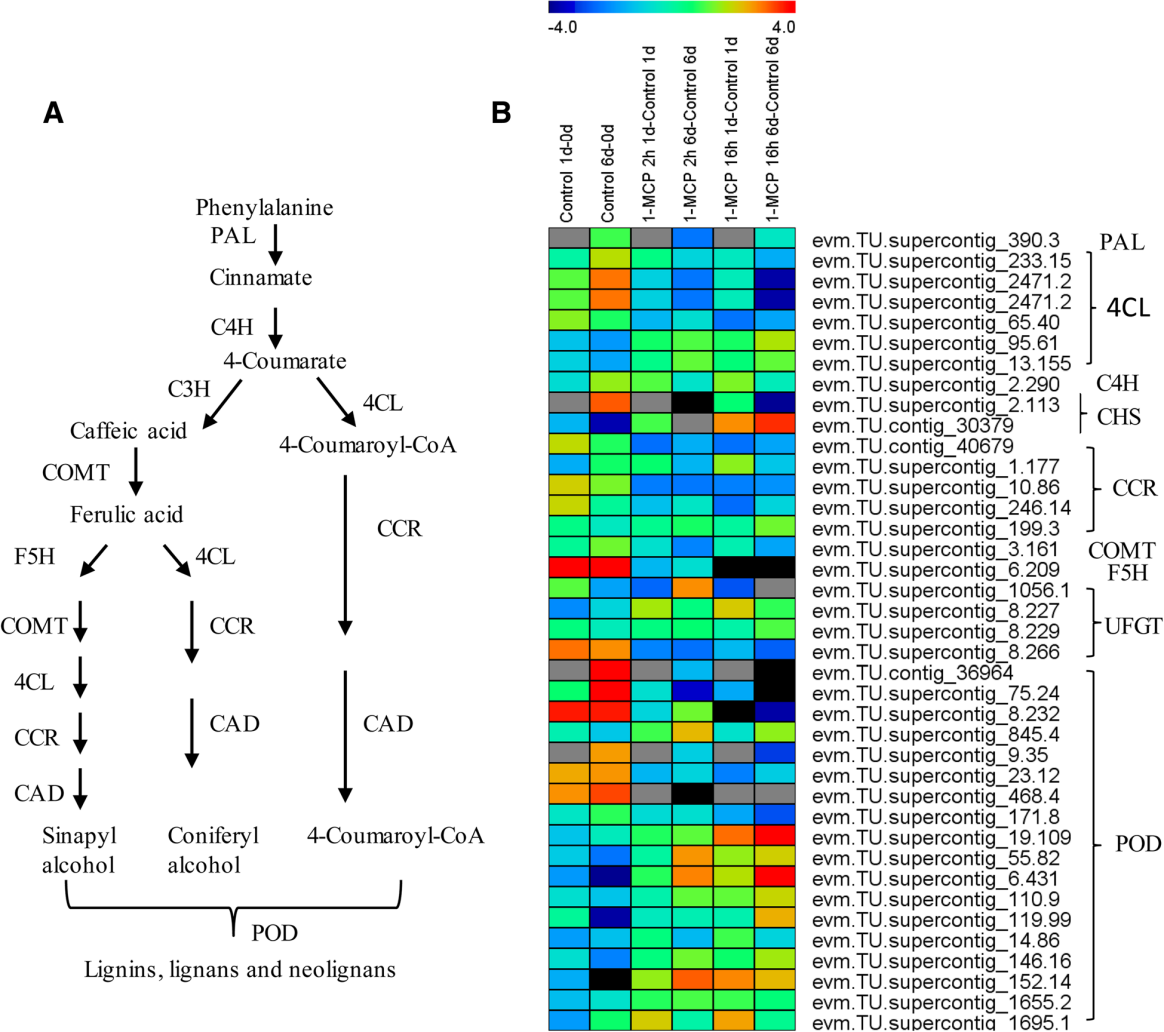
Gene expression profiles in the lignin biosynthesis pathway under 1-MCP treatments and control condition. **a**, Diagram of enzymatic steps in pathways committed to phenylpropanoid metabolism. **b**, Heat map showing genes expression profiles in lignin metabolism pathway. Abbreviations: PAL, phenylalanine ammonium lyase; 4CL, 4-coumarate-CoA ligase; C4H, cinnamate-4-hydroxylase; CHS, chalcone synthase; C3H, coumaroyl-quinate/shikimate 3-hydroxylase; COMT, caffeic acid:5-hydroxyferulic acid O-methyltransferase; CCR, cinnamoyl-CoA reductase; CAD, cinnamyl alcohol dehydrogenase; F5H, ferulate 5-hydroxylase; UFGT, UDP-glucose: flavonoid 3-O-glucosyltransferase, POD, Peroxidase. The heat map was generated by Multi Experiment Viewer (MEV)

During fruit ripening, more than 300 DEGs were related to cell wall structure (Table 2, Additional file 10: Data S1), and GO and KEGG analyses showed that most cell wall metabolism pathways are most enriched. 1-MCP treatments delayed fruit softening and affected the expression of cell wall metabolism-related genes (Additional file 12: Data S3). Transcriptome analysis between short-term and long-term 1-MCP treatments showed that a large number of DEGs were enriched in cell wall metabolism term and pathways (Fig. 4, Additional file 12: Data S3). Figure 6 shows the expression profiles of genes in lignin biosynthesis pathway, including most of the key players in lignin biosynthesis (Fig. 6a). The expression of some gene increased with fruit ripening, such as *4CL* (evm.TU.supercontig_233.15 (abbr. 233.15), 2471.2, 65.40), *CHS* (2.113), *CCR* (40,679, 10.86, 246.14), *COMT* (3.161), *F5H* (6.209), UFGT (8.266), and *POD* (8.232, 9.35, 23.12, 468.4). However, all of these genes were significantly downregulated under 1-MCP treatments, especially by long-term 1-MCP treatment (Fig. 6b). To the contrary, the expression levels of other genes were decreased with fruit ripening but induced by 1-MCP treatments, including other *4CL* (95.61, 13.155), CHS (30379), *COMT* (3.161), *UFGT* (8.227), and 10 *POD* (Fig. 6b).

**Fig. 7:**
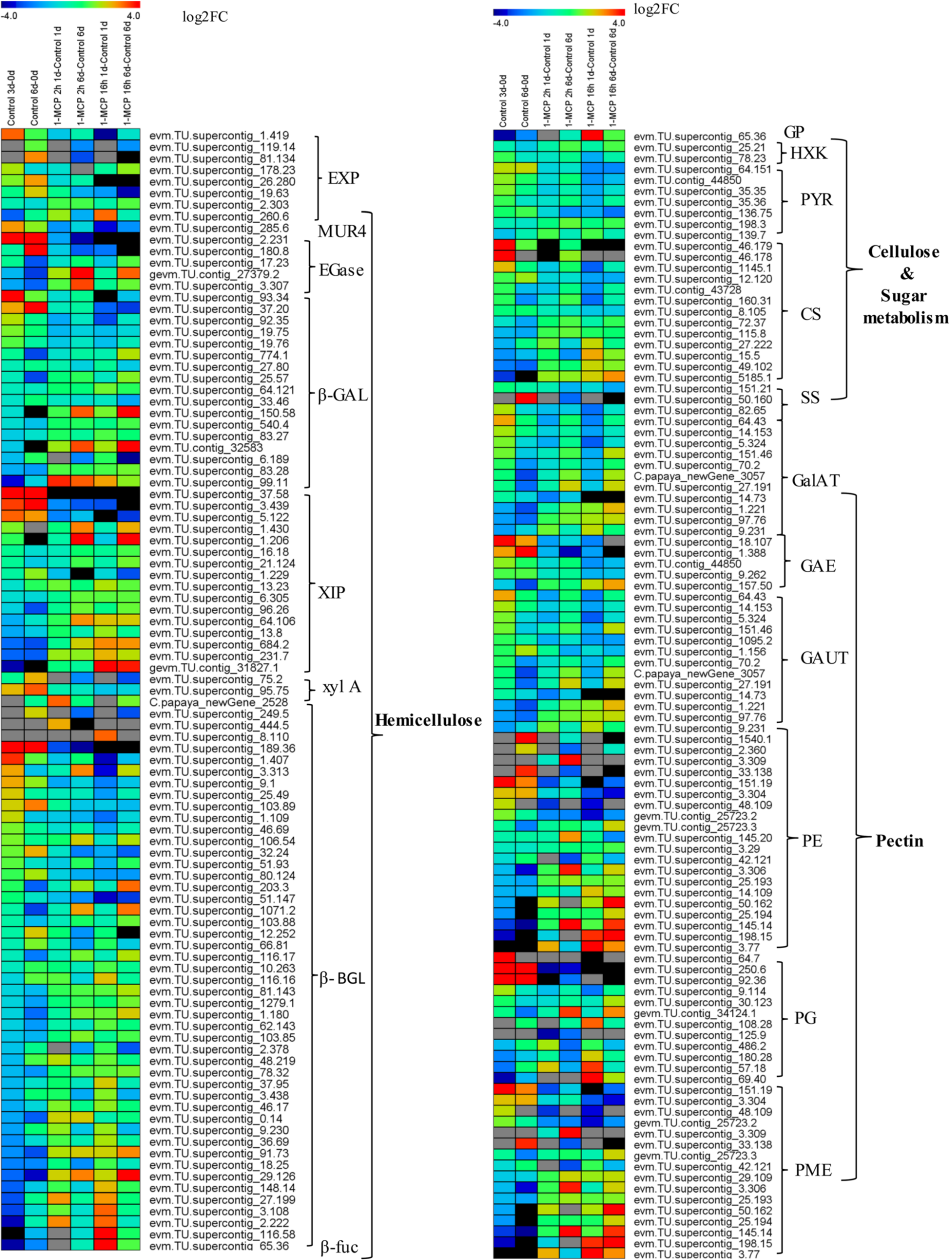
Heat map display of DEGs involved in cell wall metabolic pathways under 1-MCP treatment. Different group of DEGs were screened from the total DEGs database, which classified into the cellulose biosynthesis pathway, hemicellulose metabolic pathway, and pectin metabolic pathways. Abbreviations: EXP, expansin; MUR4, UDP-D-xylose 4-epimerase; Egase, endoglucanase; XIP, Xylanase inhibitor; β-GAL, β-galactosidase; xyl A, Xylose isomerase; BGL, β-Glucosidase; β-fuc, β-fucosidase; GP, glycogen phosphorylase; HXK, hexokinase; PYR, pyrophosphorylase; CS, cellulose synthase; SS, sucrose synthase; GalAT, galacturonosyltransferase; GAE, UDP-glucuronate-4-epimerase; GAUT, alpha-1,4-galacturonosyltransferase; PE, pectinesterase; PG, polygalacturonase; PME, pectin methylesterase. In the heat map, different color indicated the expression level changes compared with the corresponding control. The heat map was generated by Multi Experiment Viewer (MEV)

Similar to the genes involved in lignin metabolism, some genes involved in cellulose, hemicellulose, and pectin metabolism were dramatically increased during fruit ripening but repressed by 1-MCP treatments. For examples, the transcript level of *EXY1*, 5 *EXP*, 1 *MUR4*, 2 *EGase*, 5 *β-GAL*, 4 *XIP*, 2 *xyl A*, and 11 *β-BGL* genes in hemicelluloses metabolism increased with fruit ripening, but were severely repressed by 1-MCP treatments, especially for *EXY1*(106.45), *EXP* (1.419, 19.63, 26.280), *MUR4* (285.6), *EGase* (180.8, 2.231), *β-GAL* (37.20, 93.34), *XIP* (3.439, 37.58, 5.122), and *β-GAL* (1.407, 189.36) (Figs. 6 and 7). Other genes showed the opposite expression patterns, which were downregulated with fruit ripening, and induced by 1-MCP treatment, especially for *EGase* (27,379.2, 3.307), *β-GAL* (150.58, 32,583, 99.11), *XIP* (684.2, 231.7, 31,827.1), and *β-GAL* (29.126, 3.108, 116.58, 2.222) (Fig. 7). For the cellulose pathway, the expression levels of most *PYR*, 4 *CS,* and 2 *SS* genes increased with fruit ripening and were reduced by 1-MCP treatments. The expression levels of *GP, 2 PYR,* and 6 *CS* decreased with fruit ripening, but were upregulated by 1-MCP treatments (Fig. 7). For pectin metabolic pathway, the expression levels of 4 *GalAT*, 4 *GAE*, 4 *GAUT*, 7 *PE*, 4 *PG*, and 4 *PME* were repressed by 1-MCP treatments. The expression levels of other genes, including 1 *GP*, 6 *CS*, 5 *GalAT*, 1 *GAE*, 4 *GAUT*, 10 *PE,* and 9 *PME,* were induced by 1-MPC treatments (Fig. 7).

### Verification of DEGs by RT-qPCR

**Fig. 8:**
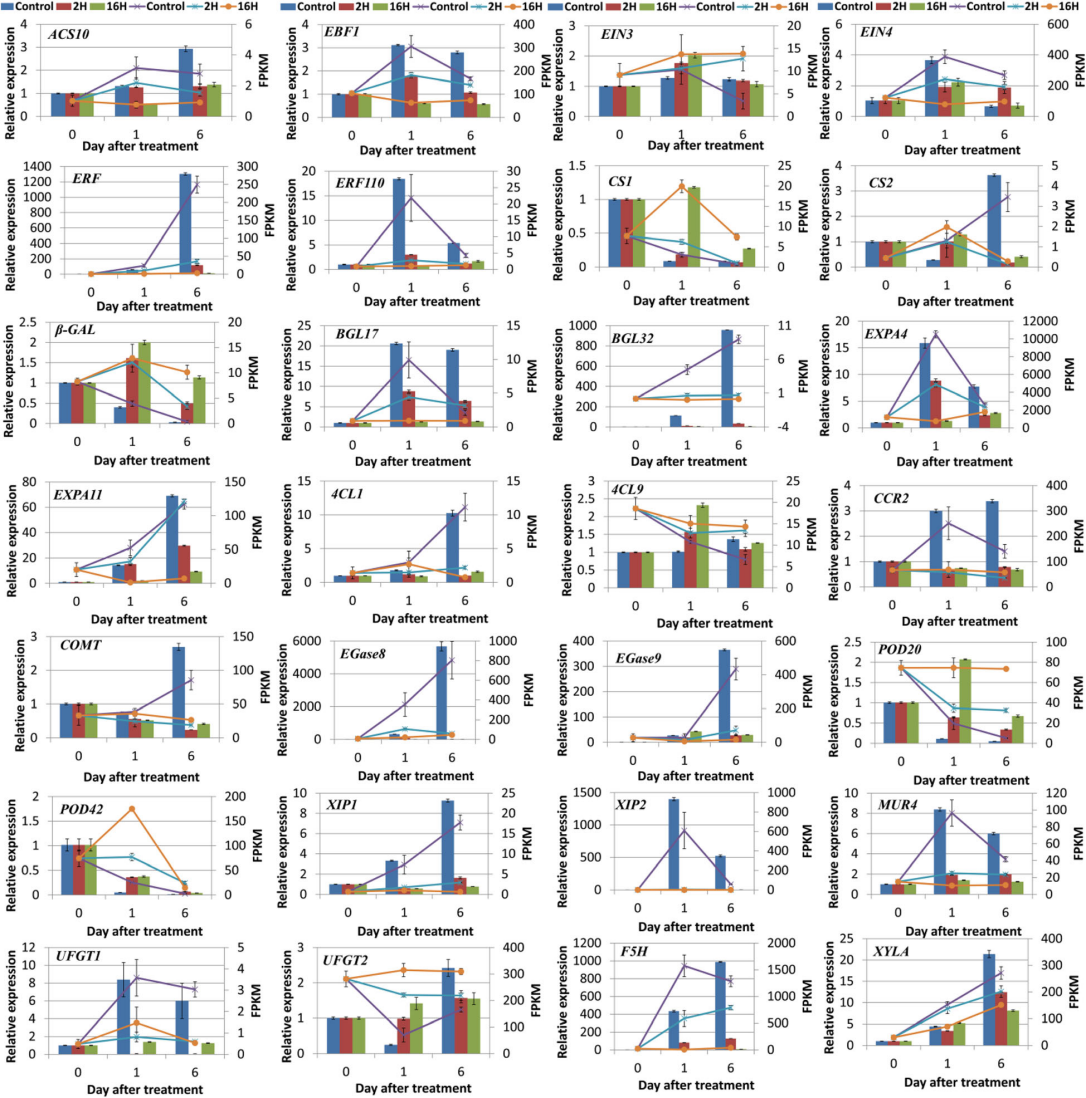
Expression pattern validation of 28 selected DEGs in the RNA-Seq analysis by qRT-PCR in papaya after 1-MCP treatments. The histograms were plotted using data obtained by RT-qPCR and the corresponded line chart was plotted by FPKM values in the RNA-seq analysis. Different colors indicate different samples

Figure 8 shows that although the fold-changes in transcript levels determined by qRT-PCR and RNA-Seq did not match exactly, the expression patterns from the two platforms were largely consistent in that the expression trends were quite similar for all of the 28 genes between the qRT-PCR analyses RNA-Seq results (Fig. 8). These results indicated that the expression profiles of these representative genes tested in the RNA-seq assay coincided with the results of RT-qPCR analysis (Fig. 8). For the six genes involved in ethylene signal pathway, *ACS10, EBF1, EIN4, ERF* and *ERF110* are positively related with fruit ripening, whose expression increased with fruit ripening. Among them, *ERF* and *ERF110* shows sharp increasing with fruit ripening, which more than1000-fold and 15-fold increasing for *ERF* and *ERF110*, respectively. ERF110 showed more rapidly response which increased dramatically at 1 DAT. However, *ERF* sharply increased at 6 DAT, which may related to fruit senescence regulation. 1-MCP treatments severely repressed their expression, especially for the long-term 1-MCP treatment. The expression of *EIN3* kept at stable level during fruit ripening, but 1-MCP treatments induced its expression. For the 22 genes involved in cell wall metabolism, among them, the expression of *BGL17, BGL32, EXPA4, EXPA11,4CL1, EGase 8, EGase 9, XIP1,XIP2, MUR4, F5H, XYLA* increased sharply with fruit ripening, but severely repressed by 1-MCP treatments, and long-term 1-MCP showed more severe repression than short-term 1-MCP treatment, indicating these genes are important for fruit ripening and closely related to fruit ripening disorder caused by long-term 1-MCP. The expression of *β-GAL,POD20* and *POD42* showed negative relationship with fruit ripening, which decreased with fruit ripening and induced by 1-MCP treatments (Fig. 8).

### Cellulose and lignin changes during fruit ripening

**Fig. 9:**
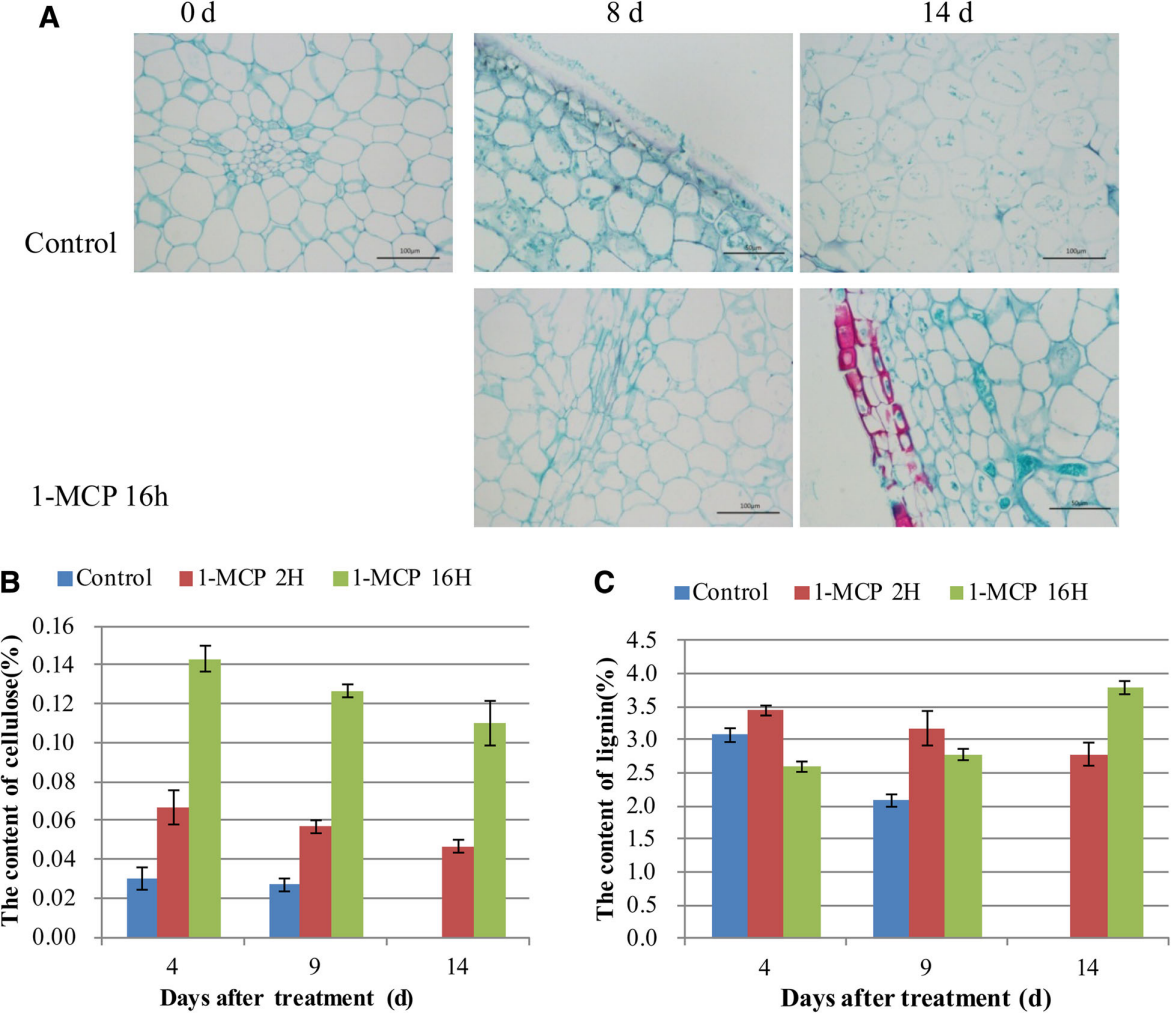
Effect of 1-MCP treatment on the lignin and cellulose content of papaya fruit. **a**, cellulose and lignin content by paraffin section observation. Safran was used to dye lignin and solid green was used to dye cellulose. Bar indicated 100 μM. **b**-**c**, the determination of cellulose (**b**) and lignin (**c**) during fruit ripening. Three biological replicates were analyzed and vertical bars indicate the SE

Paraffin sections clearly visualized changes in lignin and cellulose during fruit ripening (Fig. 9a). Cellulose was evenly distributed on the cell wall and trace amounts of lignin were observed (Fig. 9a). After four days of storage of the control fruit, cellulose, and lignin content slightly increased but cellulose content dramatically decreased at eighth day. No significant changes of cellulose and lignin observed for 1-MCP treated fruit at fourth day, but dramatically increased at the eighth day (Fig. 9a). The results were also confirmed by the determination of lignin and cellulose (Fig. 9b and c). The content of cellulose was significantly higher in the 1-MCP-treated fruit than the control (Fig. 9b). Lignin content in the control fruits slightly decreased with fruit ripening, but it increased under 1-MCP treatments (Fig. 9c). These results suggest that 1-MCP promotes the accumulation of cellulose and lignin and inhibits their degradation during fruit ripening.

## Discussion

Plant hormones are essential to fruit ripening. Fruit ripening involves complicated changes in texture, sugar, color, aroma, and flavor, and most of these events are controlled by plant hormones. Present work revealed that a large number of genes were differently expressed (FC ≥ two-fold) during fruit ripening compared to freshly harvested fruits, and carbon metabolism was the most enriched pathway by KEGG analysis. Plant hormone signal transduction, amino acids biosynthesis, and starch and sucrose metabolism were also the most enriched pathways during fruit ripening, indicating that these processes are involved in fruit ripening. It is well known that ethylene plays a major role in the ripening process of climacteric fruits [3, 9]. Three *ERF*s were significantly repressed during fruit ripening, and another four *ERF*s dramatically increased during fruit ripening in present work. More genes involved in auxin transport, synthesis, and signal transduction were identified during fruit ripening (Table 2). Several ABA receptor genes were significantly upregulated with fruit ripening. In banana and tomato fruits, ABA, IAA, and ethylene play important roles in fruit ripening [23–25]. In tomato, exogenous ABA treatment promoted ethylene synthesis and fruit ripening, whereas repressing ABA levels by the ABA biosynthesis inhibitor resulted in delayed fruit ripening and softening [24]. The contents of auxin and ABA increased during papaya fruit ripening. Thus, we propose that auxin and ABA may participate in papaya fruit ripening.

For climacteric fruits, such as tomato, banana, peach, kiwifruit, papaya, and apple, ethylene plays an important role in coordinating the physiological modifications during the ripening [3]. Acting as the inhibitor of ethylene perception, 1-MCP has drawn increasing attention. 1-MCP works on a broad range of fruits, vegetables, and floriculture crops for preventing the ethylene effect and serves as a useful tool to extend the shelf life and improve the quality of fruits, such as plum [26], tomato [17], kiwifruit [12], apple [15, 20], banana [14, 18], and papaya [5, 6]]. However, several studies showed that 1-MCP treatment may have adversely effects on fruit quality. For example, 1-MCP treatment caused flesh browning in unstored apricot fruits [27], in stored peaches [28], and nectarines [29]. It appears that 1-MCP increases storage flesh disorders. Unsuitable 1-MCP treatment also causes rubber pulp in papaya fruit [6, 7, 30] . In the present study, short-term 1-MCP delayed the ethylene peak and repressed ethylene production, delayed the fruit softening, and maintained fruit quality, which was consistent with previous studies on papaya [5]. Comparative transcriptome analysis showed that a large number of DEGs were identified between the control group and 1-MCP treatments. A lot of genes involved in fruit ripening were repressed by 1-MCP treatments, especially those genes involved in the hormone pathway and cell wall metabolism. However, the respiration peak was nearly abolished due to long-term 1-MCP treatment, resulting in rubbery pulp. The absence of climacteric may result in undesirable hardness in the pulp. Transcriptomic analyses showed that the cell wall metabolism and hormone signaling pathways were most enriched and might be important papaya fruit ripening disorder.

Ethylene was strongly reduced by 1-MCP treatment in apple, accompanied by the delayed ripening phenotype [15]. However, the effects of 1-MCP treatments on peaches and nectarines are only limited to the incubation period [29]. Fruit ripening recovered rapidly after removed from the treatment in both peaches and nectarines [28]. Ethylene biosynthesis is strongly inhibited by 1-MCP treatment in apples, but its production in peaches was not reduced by 1-MCP [15, 29]. These results indicate that the effects of 1-MCP treatment may be dependent on the fruit ripening stage, fruit cultivar, and the turnover of the ethylene receptors [15, 29]. It has been proposed that the recovery of ethylene sensitivity is mainly due to the appearance of new receptors after 1-MCP treatment [15]. For example, the transcription levels of two receptor genes, *ETR1* and *ERS1,* were almost not affected by 1-MCP and *ETR2* expression recovered when treated fruit were transferred to air [31]. 1-MCP application on fruits can mimic the ethylene binding deficiency of never ripening mutant of tomato (*Nr*). In our case, long-term 1-MCP treatment caused the failure in papaya fruit softening, which was similar to the ripening mutant. In apple, the interference of ethylene action at the receptor level by 1-MCP treatment significantly repressed a group of genes involved in the ethylene signal pathway (*ACS, ACO, ETR,* and *ERF*) [32]. Genes involved in ethylene biosynthesis and perception also showed different expression patterns in peach fruit. *ACO1* and *ETR2* were repressed by 1-MCP treatment, but *ACS1, ETR1,* and *ERS1* were not or slightly affected by 1-MCP in peach [28]. In apple, *ACS1* but not *ACO1*, was suppressed by 1-MCP treatment [32]. About 61.8% of the DEGs between control and 1-MCP treated samples were down-regulated in apple, and another 38.2% of the DEG set were positively regulated by 1-MCP, most of which are transcription factors (*ERF, NAC, MADS*, and *AUX/IAA*) and protein factors involved in the chlorophyll machinery [32].

Cell wall degradation is important trait for fruit quality, which is differentially regulated during the fruit development and the ripening process. Previous work reported that more than 50 cell wall structure-related genes are expressed during fruit development in tomato [33]. Most of the genes in the cell wall metabolsim were described by Paull et.al [34]. For example, expansins protein are important for fruit softening, and at least 15 *CpEXPA*, three *CpEXPB* and one *CpEXPLA* were isolated from papaya genome, and four of them increased with fruit ripening [34, 35]. Five SS were isolated, different gene numbers of lignin synthesis also isolated from papaya genome [34]. Notably, in the present study, more than 136 DEGs that are involved in cell-wall metabolism were identified, including cellulose synthesis, sucrose metabolism, pectin metabolism, hemicellulose metabolism, and lignin metabolism, and most of them dramatically increased during fruit ripening. Cell wall thickness and strength are key components in the maintenance of fruit firmness [36], and the textural changes during fruit ripening process are due to the changes in cell components and structure, especially those directly impacting cell wall thickness and strength. In the present work, the integrity of the cell wall was well maintained in the long-term 1-MCP treatment, which helps to maintain the abnormal fruit firmness and lead to the softening failure. Therefore, cell wall metabolism may be the key point for the fruit ripening disorder caused by 1-MCP.

Cell wall metabolism is one of the major events during fruit ripening, and diverse cell wall-modifying proteins, including enzymes for cellulose and pectin catabolism, are involved in the dismantling of these multiple polysaccharide networks. Carbohydrates play an important role in the fruit ripening process, leading to the reduced molecular size and the increasing ripening levels [37]. Starch, cellulose, pectin, and hemicelluloses are the major classes of cell wall polysaccharides that dramatically change during ripening [37, 38]. A number of genes involved in the cell wall polysaccharides were significantly repressed by long-term 1-MCP treatment.

Lignin is one of the most abundant polyphenolic polymers in higher plants just after cellulose, functioning as the structural support of the cell walls, water tightness, and response to environmental stimuli [39, 40]. The activities of POD, PAL, C4H, and 4CL are positively correlated with lignin accumulation in loquat fruit [41, 42]. To date, no plant enzymes have been reported to degrade lignin due to its complicated structure in higher plants [43]. Therefore, it is important to find the effective means to inhibit lignin formation, rather than degrade or disperse it. It has been reported that several postharvest approaches, such as 1-MCP, could inhibit lignification in fruit, such as loquat [12, 44]. However, 1-MCP enhanced lignification of loquat fruit during the later storage period and induced enzymatic activities of PAL, CAD, and POD, with a higher lignification incidence and lignin content [12]. In stark contrast, the activities of PAL, CAD, and POD and lignin accumulation were reduced by 1-MCP treatment in postharvest bamboo shoots [45]. In the present study, the lignin metabolism-related gene family showed different expression patterns. Genes, including *4CL, CHS, CCR, COMT, F5H, UFGT*, and *POD,* were upregulated with fruit ripening but were significantly downregulated by 1-MCP treatments, especially by the long-term 1-MCP treatment (Fig. 6b). Other gene families, such as other members of *4CL, CHS, COMT* (3.161), *UFGT*, and *POD,* were downregulated with fruit ripening but were upregulated by 1-MCP treatments (Fig. 6b), which may be important to the rubbery pulp phenomenon.

Pectins are another group of important players that regulating the texture and quality of fruit, and they are the major components of primary cell wall and middle lamella. Pectins are degraded with fruit ripening and mainly contributed to fruit softening. Pectin-degrading enzymes, including polygalacturonase (PG), pectin methylesterase (PME), pectate lyase (PL), and rhamnogalacturonase, are closely related to fruit-tissue softening [4, 38]. Genes involved in hemicellulose, cellulose, and pectin metabolism showed the similar expression profiles to those of genes involved in lignin metabolism (Fig. 7). For example, some genes were dramatically increased during fruit ripening but repressed by 1-MCP treatment, including *endoxylanase (EXP1)*, 5 *EXP*, 1 *MUR4*, 2 *EGase*, 5 *β-GAL*, 4 *XIP*, 2 *xyl A*, and 11 *β-BGL* genes in hemicelluloses (Fig. 7). Especially for *EXP1*, which dramatically increased at 1 DAT (more than 2000-fold of 0 DAT) and 6DAT, but severely repressed by 1-MCP treatments, and no expression detected in long-term 1-MCP treated fruit (Fig. 5, Additional file 9: Table S5). It was well documented that endoxylanase play an important roles in fruit softening, which expressed with fruit ripening and softening [7, 46]. Previous work also showed that 1-MCP-treated fruit with ‘rubbery’ texture showed suppressed endoxylanase gene expression, protein and enzymatic activity [7]. All these results indicated that endoxylanase may play important roles in 1-MCP induced ripening disorder. Other genes showed the opposite expression patterns, which decreased with fruit ripening, and they were induced by 1-MCP treatments. Similar results also observed in apple fruit treated with 1-MCP. 1-MCP treatment repressed a majority of genes encoding cell wall enzymes (50 of 77), such as pectinesterase, polygalacturonase, and *PL*, and they also induced a specific group of cell wall-related genes [32]. Additionally, the degradation pectin and cellulose usually depends on ethylene production during the ripening of climacteric fruits [37, 38]. A close relationship exists between endo-PG and ethylene production during peach fruit ripening, which marked reduction of *endo-PG* expression in 1-MCP treated fruit and severe inhibition of ethylene production [27]. *PpEXP2* and *PpEXP2* showed opposite expression patterns during fruit ripening and 1-MCP treatment [27]. In papaya fruit, The activities of hydrolases including endoglucanase, β-galactosidase, PME, β-xylosidase and endoxylanase were dramatically increased with fruit ripening, but 1-MCP severely repressed their activities, which may correlated “rubbery” texture caused by 1-MCP [8]. Our previous work also showed that the activities of corresponding key enzymes for cell wall degradation in papaya, including PME, PG, PL, and cellulase (CX), were also dramatically repressed by 1-MCP in papaya fruit [30]. These enzymes play important roles in cell wall degradation, in an coordinated and interdependent manner [38, 47].

The content of two key components of the cell wall, cellulose and lignin, were relatively higher in 1-MCP-treated fruits than the controls during storage, which may account for the rubbery texture caused by long-term 1-MCP treatment.

## Conclusions

**Fig. 10:**
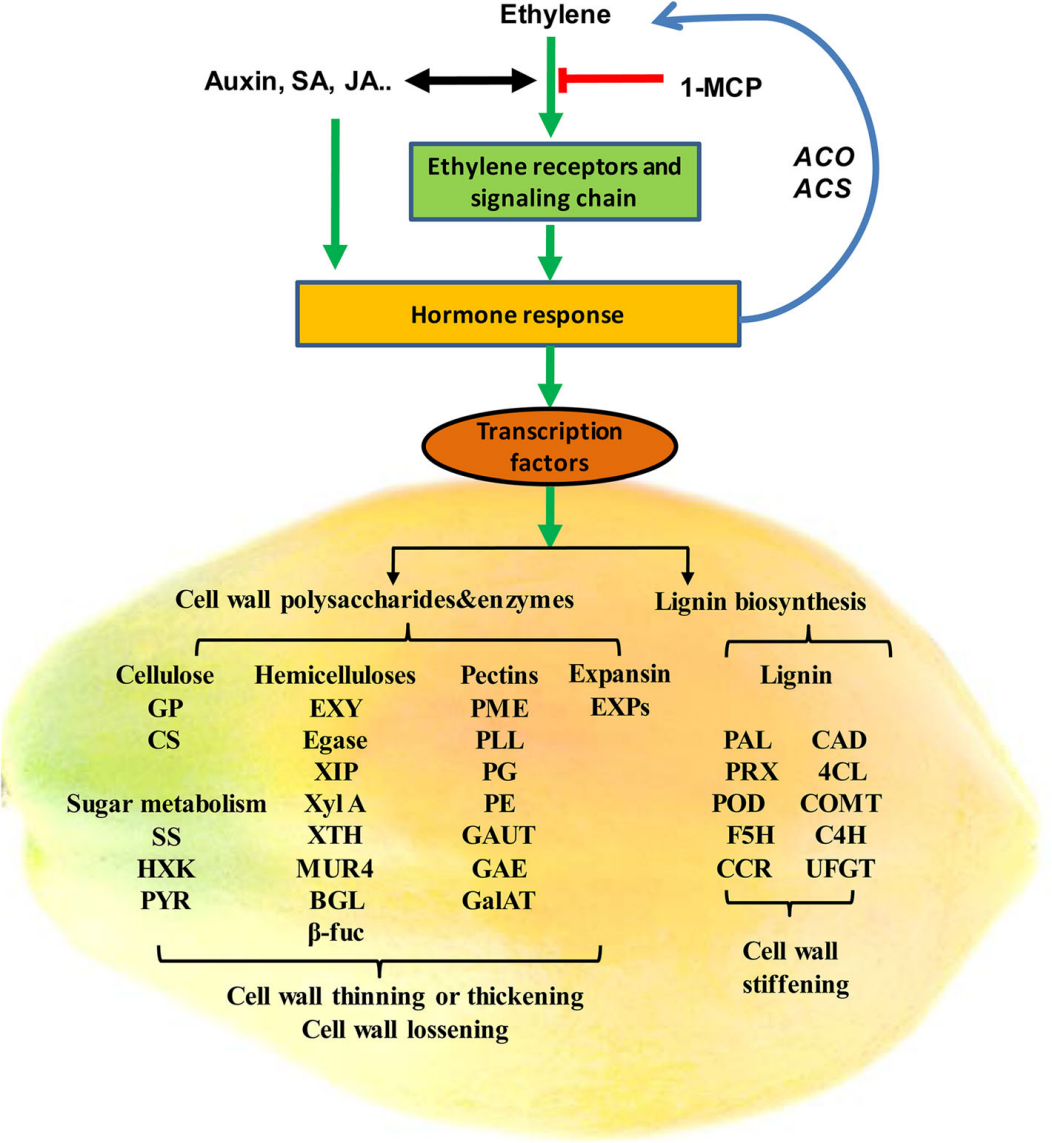
Diagram summarizing the plant cell wall response to 1-MCP treatments. Abbreviations: *SS, sucrose synthase;* GP, glycogen phosphorylase; HXK, hexokinase; PYR, pyrophosphorylase; EXY, endoxylanase; CS, cellulose synthase; Egase, endoglucanase; β-GAL, β-galactosidase; EXP, expansin; XIP, Xylanase inhibitor; xyl A, Xylose isomerase; XTH, xyloglucan endo-β-transglucosylases/hydrolases; MUR4, UDP-D-xylose 4-epimerase; BGL, β-Glucosidase; β-fuc, β-fucosidase; PME, pectin methylesterase; PLL, pectin/pectate lyase-like; PG, polygalacturonase; *PE, pectinesterase;* GAUT, alpha-1,4-galacturonosyltransferase; GAE, UDP-glucuronate-4-epimerase; GalAT, galacturonosyltransferase; PAL, phenylalanine ammonium lyase; CAD, cinnamyl alcohol dehydrogenase; 4CL, 4-coumarate-CoA ligase; POD, Peroxidase; COMT, caffeic acid:5-hydroxyferulic acid O-methyltransferase; F5H, ferulate 5-hydroxylase; C4H, cinnamate-4-hydroxylase; CHS, chalcone synthase; CCR, cinnamoyl-CoA reductase; UFGT, UDP-glucose: flavonoid 3-O-glucosyltransferase; ACS, ACC Synthase; ACO, ACC Oxidase

1-MCP treatment provides enormous information on fruit ripening and ripening disorder caused by long-term 1-MCP application, especially fruit softening. A summary of the cell wall responses to 1-MCP treatment is shown in Fig. 10. Suitable 1-MCP treatment reduced ethylene production and cell wall polysaccharides and enzymes activities, and it affected the lignin biosynthesis pathway, which in turn delayed fruit softening. However, long-term 1-MCP treatment severely inhibited the ethylene biosynthesis and signal transduction pathway, and then it resulted in the inhibition of cell wall enzymes and polysaccharides activities, enhancing the lignin biosynthesis pathway. These actions effectively inhibited cell wall thinning and cell wall loosening, and enhanced the cell wall stiffening, which prevented fruit softening.

## Methods

### Plant materials and treatments

Papaya fruits (*Carica papaya* L., cv. Suiyou-2′) of color break stage (5% < peer color < 15% yellow) were harvested from a local commercial farm in Panyu District of Guangzhou, Guangdong, South China. The fruit with similar size and free of blemishes were selected, first washed with water, dipped in to 0.2% (w/v) hypochloride solution for 10 min and then soaked in 500 mg•mL^− 1^ mixture solutions of iprodione (Kuaida, Jiangsu, China) and prochloraz (Huifeng, Jiangsu, China) for 1 min to eliminate potential microbes. After being air-dried at 22 °C, three different treatments were performed, namely, 400 nL•L^− 1^ of 1-MCP for 16 h (long-term 1-MCP), 400 nL•L^− 1^ of 1-MCP for 2 h (short-term 1-MCP), and the control group with 0 nL•L^− 1^ of 1-MCP treatment. Then all of the fruits were soaked in 1000 μL•L^− 1^ ethephon solution for 1 min and placed into unsealed plastic bags (0.02 mm thick) for ripening at 25 °C. For the control treatment, samples were taken at 0, 1, 2, 4, and 6 days. For both 1-MCP treatments, samples were taken at 0, 1, 2, 4, 6, 8, 11, and 14 days after treatment (DAT). Samples were frozen in liquid nitrogen and then stored at − 80 °C. All of the treatments were conducted with three biological replicates.

### Fruit firmness, respiration, ethylene production, and coloring index assessment

Fruit firmness, respiration, ethylene production, and coloring index assessment were determined as described by Li et al. [48].

### The content determination of cellulose and lignin

The cellulose content were determined as described by Niu S [49], and lignin content was determined as described by Ermakov et al. [50].

### Ultrastructure and microstructure observations of the fruit cell walls

Fruit tissues from different storage periods were taken and fixed with 2.5% glutaraldehyde and 1% osmium tetroxide, then treated as follows: they were washed with PBS (phosphate buffer saline) buffer, eluted with an ethanol gradient, embedded in SPI812 resin, ultrasonically sectioned on an Ultracut Uct (Leica, Germany), and stained with uranyl acetate and lead citrate. Images were captured using a transmission electron microscopy TECNAI-12 (PHILIPS, Holland) using a 2KV accelerating voltage.

For observing the microstructure of the fruit cells, pieces of fruits pulp (0.5 cm × 0.5 cm × 0.5 cm) were taken, fixed, and then sectioned with a freezing microtome set to 20-μm thickness. The thin layer samples were visualized and analyzed under a fluorescence microscope (Axioskop Plus, Germany).

### cDNA library preparation and Illumina sequencing

Total RNAs were extracted from fruit pulp samples using PureLink^R^ Plant RNA Reagent (Ambion, #12322–012), according to the manufacturer’s protocol. RNA concentration was measured using NanoDrop 2000 (Thermo). RNA integrity was examined using an RNA Nano 6000 Assay Kit and the Agilent Bioanalyzer 2100 system (Agilent Technologies, Santa Clara, CA, USA). RNA-Seq libraries were generated using NEB Next UltraTM RNA Library Prep Kit for Illumina (NEB, #E7530, USA) following manufacturer’s recommendations, and index codes were added to attribute sequences to each sample. Fruit samples in the control, short-term and long-term 1-MCP treatment groups after 0, 1, and 6 days of storage were selected for transcriptomic analysis. Each sample time point contained three biological replicates, and a total of 21 libraries were constructed. All of the libraries were sequenced on an Illumina Hiseq Xten platform by the Biomarker Technology Company (Beijing, China).

### Sequence assembly and functional annotation

Clean reads were mapped to the reference genome of papaya (http://www.plantgdb.org/CpGDB/) using TopHat2 Software. The reads of each biological replicate were mapped independently, and only reads with a perfect match or one mismatch were further analyzed and annotated based on the reference genome. Only uniquely mapped reads were used in the subsequent analysis of gene expression profiles in different treatment groups. Sequences were aligned using BLASTx to non-redundant protein databases (Nr) of the National Center for Biotechnology Information (NCBI). Gene annotation was performed using various methods, which were as follows: Swiss-Prot protein databases, COG, KOG, and KEGG. Coding sequences (CDS) of genes were predicted by Trans Decoder Software (http://transdecoder.github.io).

### Analysis of differentially expressed genes (DEG)

Gene expression levels were estimated using the fragments per kilobase of the transcript per million mapped reads (FPKM) method [51]. DEGs were identified using the DESeq Software [52] in pair-wise comparisons. The results of all of the statistical tests were revised to account for multiple testing with the Benjamini-Hochberg approach for controlling the false discovery rate (FDR < 0.05). Genes were determined to be significantly and differentially expressed at a *P* value (< 0.05), and Fold change (FC) > 2. Gene Ontology (GO) enrichment analysis of DEGs was conducted using the GOseq R packages based Wallenius non-central hyper-geometric distribution [53]. KOBAS [54] software was used to test the statistical enrichment of DEGs in the KEGG pathways.

### Validation of DEGs by qRT-PCR

Twenty-eight genes involved in plant hormone signal and cell wall metabolism pathway were selected for further validation by RT-qPCR. Transcript levels of 6 genes are involved plant hormone synthesis and signal transduction, and 22 are involved in cell wall metabolism, including the lignin metabolism, cellulose biosynthesis pathway, hemicellulose metabolic pathway, and pectin metabolic pathways. Gene expression analyses were performed using the quantitative real-time PCR analysis (RT-qPCR). Total RNA was extracted using hot borate method [55]. RT-qPCR was performed according to our previously optimized methods [56]. The primers of all of the genes tested were listed in Additional file 5: Table S1. *CpTBP1* and *CpACTIN* were selected as reference genes in papaya as validated previously [56]. Three technical replicates were included each biological replicate. The analysis of variance (ANOVA) was based on Duncan’s multiple range test (DMRT) in SPSS 19.0 (IBM, USA).

## Additional files

Additional file 1: **Figure S1.** Overview of papaya transcriptomes of fruit treated with or without 1-MCP treatment. (A), Pairwise correlation of different biological replicates from control fruit and 1-MCP-treated fruit using FPKM values. The color intensities (scale in the side bar) and the numbers indicate the degree of pairwise correlation. (B), Gene expression level (log10 FPKM) of all of the samples. The expression level of each sample from the overall dispersion of the expression volume. (C), GO classification of assembled *Carica papaya*. The results were summarized in three main GO categories: cellular component, molecular function, and biological process. The right y-axis indicated the number of the assembled unigenes and DEGs. (TIF 4007 kb)

Additional file 2: **Figure S2.** Sequences identity blast with other species (A) and COG classification of assembled *Carica papaya* unigenes. (TIF 2740 kb)

Additional file 3: **Figure S3.** Venn diagrams and Histogram of GO term of DEGs comparison of 1-MCP treatment and the control condition. A, The number of differentially expressed genes derived from comparison between the short-term 1-MCP treatment on 1 d and 6 d samples and the control sample at each time point. B, The number of differentially expressed genes derived from comparison between long-term 1-MCP treatment on 1 d and 6 d samples and the control samples at each time point. Software (http://bioinformatics.psb.ugent.be/webtools/Venn/) was used for the Venn diagram. C, Top 20 enrichment GO term of DEGs comparison of 1-MCP treatment (400, 2 h) and the control condition on 1DAT and 6DAT. D, Top 20 enrichment GO term of DEGs comparison of 1-MCP treatment (400, 16 h) and the control condition on 1 DAT and 6DAT. (TIF 3449 kb)

Additional file 4: **Figure S4.** Top 20 enriched KEGG pathways identified with KEGG Orthology-Based Annotation System (KOBAS) 2.0 in papaya fruit of DEG comparison between long-term 1-MCP treatment and short-term 1-MCP treatment after they were harvested on 1 d and 6 d. (TIF 1353 kb)

Additional file 5: **Table S1.** Primer sequences used in present study. (XLSX 14 kb)

Additional file 6: **Table S2.**. Summary of the sequencing reads and their matches in the papaya genome. (XLS 27 kb)

Additional file 7: **Table S3.** Selected DEGs involved in hormone signal pathway during fruit ripening. (XLSX 17 kb)

Additional file 8: **Table S4.** Selected DEGs involved in cell wall metabolism pathway. (XLSX 21 kb)

Additional file 9: **Table S5.** Selected DEGs involved in hormones pathway under 1-MCP treatments. (XLSX 39 kb)

Additional file 10: Data S1. Functional annotation and identification of unigenes by blast in different databases. (XLS 16696 kb)

Additional file 11: Data S2. KEGG classification of DEG during fruit ripening. (XLS 96 kb)

Additional file 12: Data S3. DEGs during fruit ripening enriched in cell wall metabolism term and pathways. (XLSX 51 kb)

## Data Availability

The datasets generated and analyzed during the current study are available in the supplemental materials and from the corresponding author on reasonable request.

## Abbreviations

1-MCP: 1-methylcyclopropene
ABA: Abscisic acid
*ACO*: ACC oxidase
*ARF*s: Auxin response factors
CH: Chloroplast
CK: Cytokinin
CW: Cell wall
DAT: Days after treatment
DEGs: Differently expressed genes
*ERFs*: Ethylene-responsive transcription factor
*ERS*: Ethylene response sensor
*EXY1*: *Endoxylanase*
FC: Fold change
GA: Gibberellin
Gb: Gigabytes
GO: Gene ontology
JA: Jasmonic acid
KEGG: Kyoto encyclopedia ofgenes and genomes
MF: Microfibrous filaments
PKM: Fragments perkilo base of transcript per million mapped reads
RNA-Seq: RNA sequencing
RT-qPCR: Quantitative reverse transcription polymerase chain reaction
SEM: Scanning electron microscopy
SG: Starch granules
TEM: Transmission electron microscope
