# Supplementary figures and images for: Transcriptomic analysis reveals key factors in fruit ripening and rubbery texture caused by 1-MCP in papaya
Source: BMC Plant Biol. 2019 Jul 12;19:309. doi: 10.1186/s12870-019-1904-x (PMC6626363; doi:10.1186/s12870-019-1904-x)

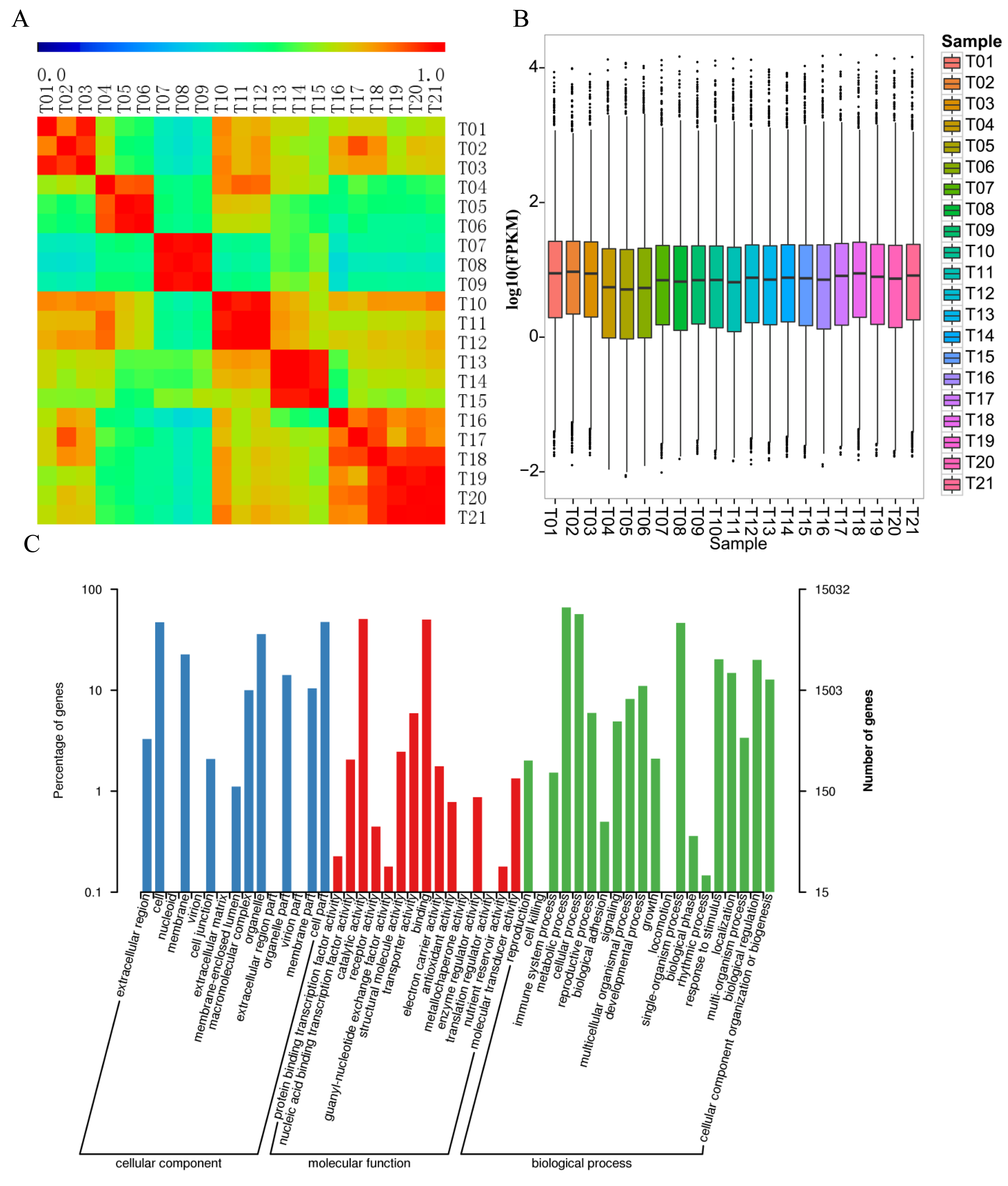

Supplement: Supplementary file 1 — Figure S1. Overview of papaya transcriptomes of fruit treated with or without 1-MCP treatment. (A), Pairwise correlation of different biological replicates from control fruit and 1-MCP-treated fruit using FPKM values. The color intensities (scale in the side bar) and the numbers indicate the degree of pairwise correlation. (B), Gene expression level (log10 FPKM) of all of the samples. The expression level of each sample from the overall dispersion of the expression volume. (C), GO classification of assembled Carica papaya. The results were summarized in three main GO categories: cellular component, molecular function, and biological process. The right y-axis indicated the number of the assembled unigenes and DEGs. (TIF 4007 kb) [file 12870_2019_1904_MOESM1_ESM.tif]

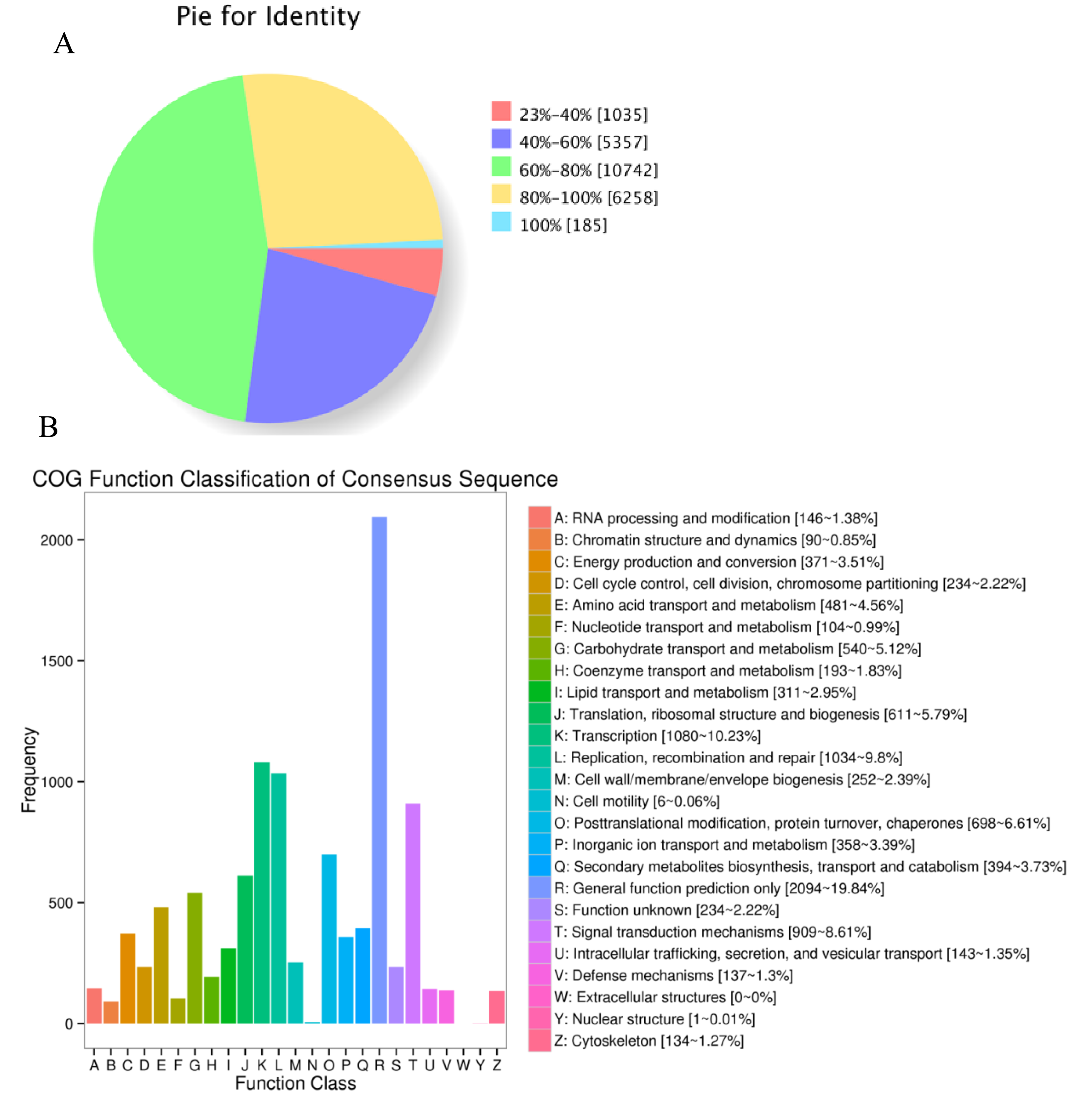

Supplement: Supplementary file 2 — Figure S2. Sequences identity blast with other species (A) and COG classification of assembled Carica papaya unigenes. (TIF 2740 kb) [file 12870_2019_1904_MOESM2_ESM.tif]

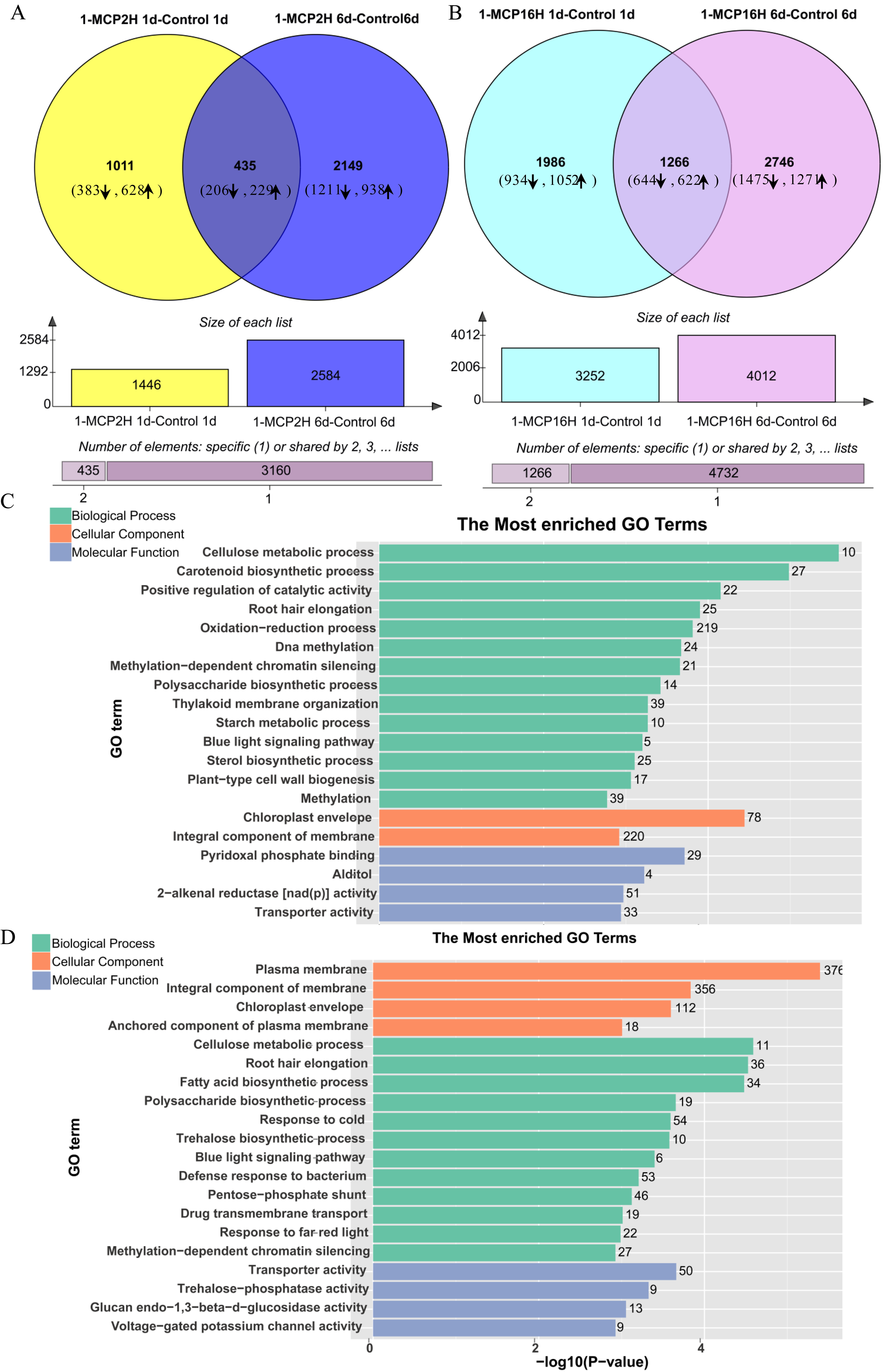

Supplement: Supplementary file 3 — Figure S3. Venn diagrams and Histogram of GO term of DEGs comparison of 1-MCP treatment and the control condition. A, The number of differentially expressed genes derived from comparison between the short-term 1-MCP treatment on 1 d and 6 d samples and the control sample at each time point. B, The number of differentially expressed genes derived from comparison between long-term 1-MCP treatment on 1 d and 6 d samples and the control samples at each time point. Software (http://bioinformatics.psb.ugent.be/webtools/Venn/) was used for the Venn diagram. C, Top 20 enrichment GO term of DEGs comparison of 1-MCP treatment (400, 2 h) and the control condition on 1DAT and 6DAT. D, Top 20 enrichment GO term of DEGs comparison of 1-MCP treatment (400, 16 h) and the control condition on 1 DAT and 6DAT. (TIF 3449 kb) [file 12870_2019_1904_MOESM3_ESM.tif]

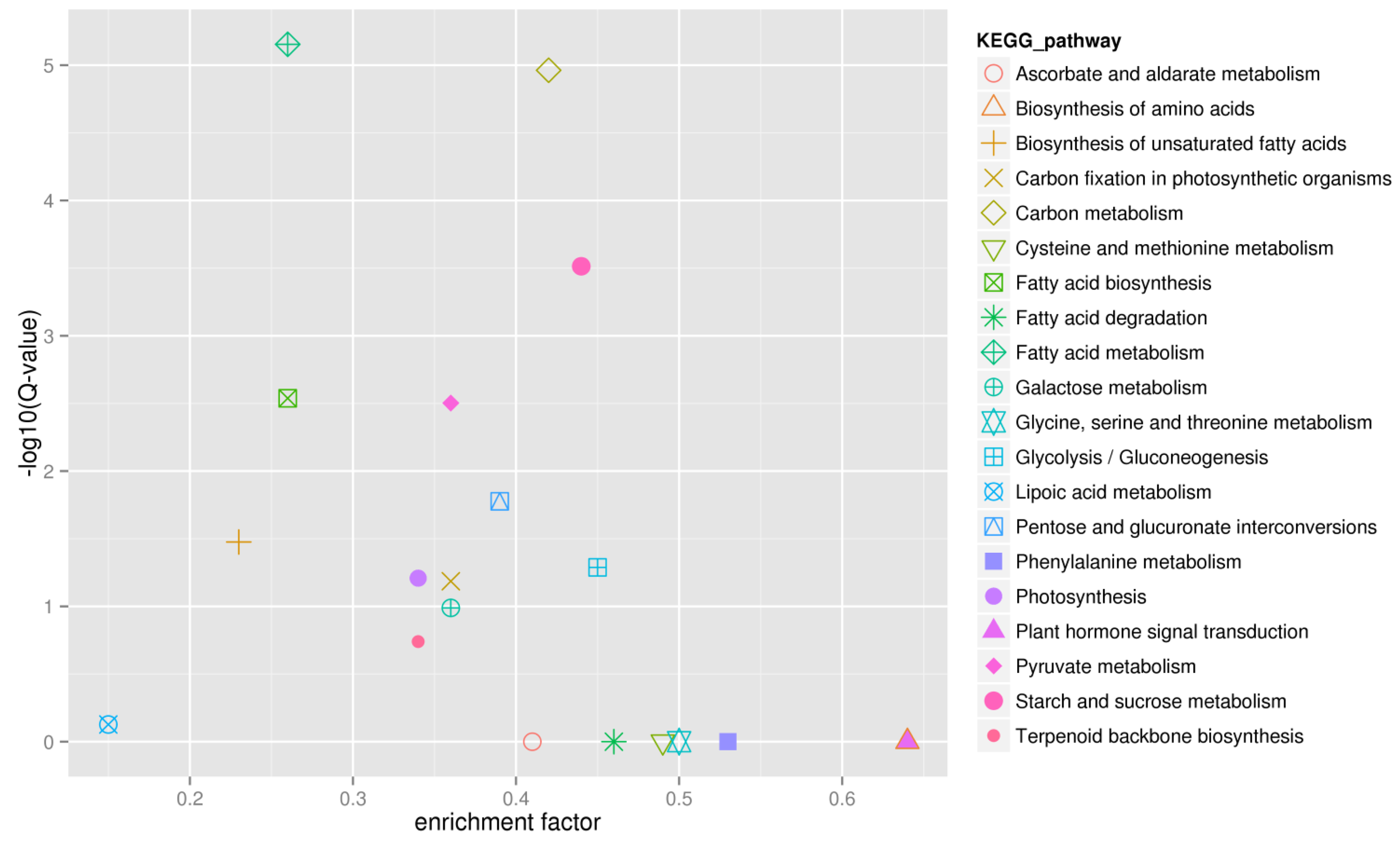

Supplement: Supplementary file 4 — Figure S4. Top 20 enriched KEGG pathways identified with KEGG Orthology-Based Annotation System (KOBAS) 2.0 in papaya fruit of DEG comparison between long-term 1-MCP treatment and short-term 1-MCP treatment after they were harvested on 1 d and 6 d. (TIF 1353 kb) [file 12870_2019_1904_MOESM4_ESM.tif]
